# Leukemia-Associated Somatic Mutations Drive Distinct Patterns of Age-Related Clonal Hemopoiesis

**DOI:** 10.1016/j.celrep.2015.02.005

**Published:** 2015-02-26

**Authors:** Thomas McKerrell, Naomi Park, Thaidy Moreno, Carolyn S. Grove, Hannes Ponstingl, Jonathan Stephens, Understanding Society Scientific Group, Charles Crawley, Jenny Craig, Mike A. Scott, Clare Hodkinson, Joanna Baxter, Roland Rad, Duncan R. Forsyth, Michael A. Quail, Eleftheria Zeggini, Willem Ouwehand, Ignacio Varela, George S. Vassiliou

**Affiliations:** 1 Haematological Cancer Genetics, Wellcome Trust Sanger Institute, Cambridge CB10 1SA, UK; 2 Sequencing Research Group, Wellcome Trust Sanger Institute, Cambridge CB10 1SA, UK; 3 Instituto de Biomedicina y Biotecnología de Cantabria (CSIC-UC-Sodercan), Departamento de Biología Molecular, Universidad de Cantabria, 39011 Santander, Spain; 4 Department of Haematology, Cambridge Biomedical Campus, University of Cambridge, Cambridge CB2 0XY, UK; 5 NHS Blood and Transplant, Cambridge Biomedical Campus, Cambridge CB2 0PT, UK; 6 Institute for Social and Economic Research, University of Essex, Colchester CO4 3SQ, UK; 7 Department of Haematology, Cambridge University Hospitals NHS Trust, Cambridge CB2 0QQ, UK; 8 Cambridge Blood and Stem Cell Biobank, Department of Haematology, University of Cambridge, Cambridge CB2 0XY, UK; 9 Department of Medicine II, Klinikum Rechts der Isar, Technische Universität München, 81675 München, Germany; 10 German Cancer Consortium (DKTK), German Cancer Research Center (DKFZ), 69120 Heidelberg, Germany; 11 Department of Medicine for the Elderly, Cambridge University Hospitals NHS Trust, Cambridge CB2 0QQ, UK; 12 Human Genetics, Wellcome Trust Sanger Institute, Cambridge CB10 1SA, UK

## Abstract

Clonal hemopoiesis driven by leukemia-associated gene mutations can occur without evidence of a blood disorder. To investigate this phenomenon, we interrogated 15 mutation hot spots in blood DNA from 4,219 individuals using ultra-deep sequencing. Using only the hot spots studied, we identified clonal hemopoiesis in 0.8% of individuals under 60, rising to 19.5% of those ≥90 years, thus predicting that clonal hemopoiesis is much more prevalent than previously realized. *DNMT3A-*R882 mutations were most common and, although their prevalence increased with age, were found in individuals as young as 25 years. By contrast, mutations affecting spliceosome genes *SF3B1* and *SRSF*2, closely associated with the myelodysplastic syndromes, were identified only in those aged >70 years, with several individuals harboring more than one such mutation. This indicates that spliceosome gene mutations drive clonal expansion under selection pressures particular to the aging hemopoietic system and explains the high incidence of clonal disorders associated with these mutations in advanced old age.

## Highlights

• Clonal hemopoiesis is an almost inevitable consequence of aging in humans
• Spliceosome gene mutations drove clonal hemopoiesis only in persons aged ≥70 years
• NPM1 mutations behave as gatekeepers for leukemogenesis

## Graphical Abstract

**Figure:**
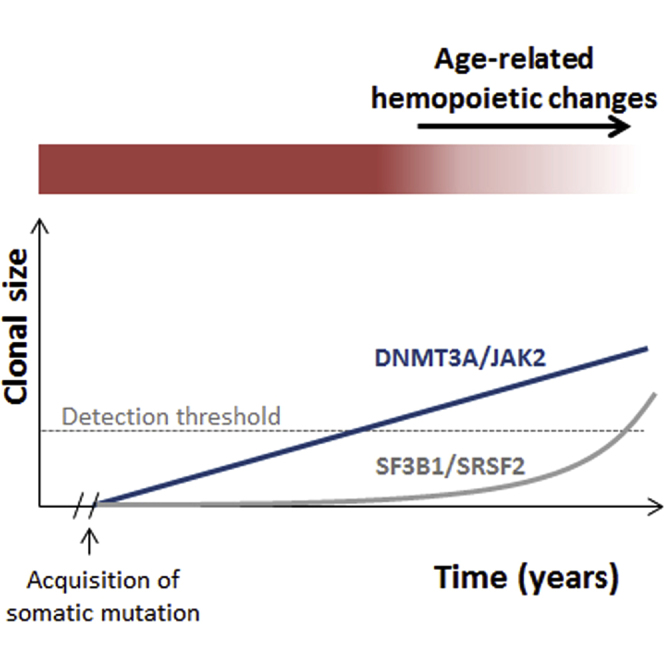

## Teaser

McKerrell et al. employ ultra-deep sequencing to show that age-related clonal hemopoiesis is much more common than previously realized. They find that clonal hemopoiesis, driven by mutations in spliceosome genes *SF3B1* and *SRSF2*, was noted exclusively in individuals aged 70 years or older and that *NPM1* mutations are not seen in association with this phenomenon, endorsing their close association with leukemogenesis.

## Introduction

Cancers develop through the combined action of multiple mutations that are acquired over time (Nowell, 1976). This paradigm is well established in hematological malignancies, whose clonal history can be traced back for several years or even decades (Ford et al., 1998; Kyle et al., 2002). It is also clear from studies of paired diagnostic-relapsed leukemia samples that recurrent disease can harbor some, but not always all, mutations present at diagnosis, providing evidence for the presence of a clone of ancestral pre-leukemic stem cells that escape therapy and give rise to relapse through the acquisition of new mutations (Ding et al., 2012; Krönke et al., 2013). Studies of such phenomena have defined a hierarchical structure among particular leukemia mutations, with some, such as those affecting the gene *DNMT3A*, displaying the characteristics of leukemia-initiating lesions and driving the expansion of hemopoietic cell clones prior to the onset of leukemia (Ding et al., 2012; Shlush et al., 2014).

These observations suggest that individuals without overt features of a hematological disorder may harbor hemopoietic cell clones carrying leukemia-associated mutations. In fact, such mutations, ranging from large chromosomal changes (Jacobs et al., 2012; Laurie et al., 2012) to nucleotide substitutions (Busque et al., 2012), have been found to drive clonal hemopoiesis in some individuals. Recent reanalyses of large exome-sequencing data sets of blood DNA showed that clonal hemopoiesis is more common than previously realized and increases with age to affect up to 11% of those over 80 and 18.4% of those over 90 years (Genovese et al., 2014; Jaiswal et al., 2014; Xie et al., 2014). The presence of such clones was associated with an increased risk of developing hematological or other cancers and a higher all-cause mortality, probably due to an increased risk of cardiovascular disease (Genovese et al., 2014; Jaiswal et al., 2014).

The important findings of these studies were based on analysis of exome-sequencing data sets that were generated for the study of constitutional genomes, thus trading genome-wide coverage for reduced sensitivity for detecting small subclonal events. We used the different approach of targeted re-sequencing of selected leukemia-associated mutation hot spots in blood DNA from more than 4,000 individuals unselected for blood disorders. In addition to increasing the sensitivity for detecting subclonal mutations, this approach enabled us to prospectively select and study a large number of elderly individuals. Our results show that clonal hemopoiesis is significantly more common than anticipated, give new insights into the distinct age-distribution and biological behavior of clonal hemopoiesis driven by different mutations, and help explain the increased incidence of myelodysplastic syndromes (MDSs) with advancing age.

## Results

To investigate the incidence, target genes, and age distribution of age-related clonal hemopoiesis (ARCH), we performed targeted re-sequencing for hot spot mutations at 15 gene loci recurrently mutated in myeloid malignancies (Table 1) using blood DNA from 3,067 blood donors aged 17–70 (Wellcome Trust Case Control Consortium [WTCCC]) and 1,152 unselected individuals aged 60–98 years (United Kingdom Household Longitudinal Study [UKHLS]; see Figure S1 for detailed age distributions). To do this, we developed and validated a robust methodology, employing barcoded multiplex PCR of mutational hot spots followed by next-generation sequencing (MiSeq) and bioinformatic analysis, to extract read counts and allelic fractions for reference and non-reference nucleotides. This reliably detected mutation-associated circulating blood cell clones with a variant allele fraction (VAF) ≥ 0.008 (0.8%; see Supplemental Experimental Procedures and Figure S2).

**Figure 1:**
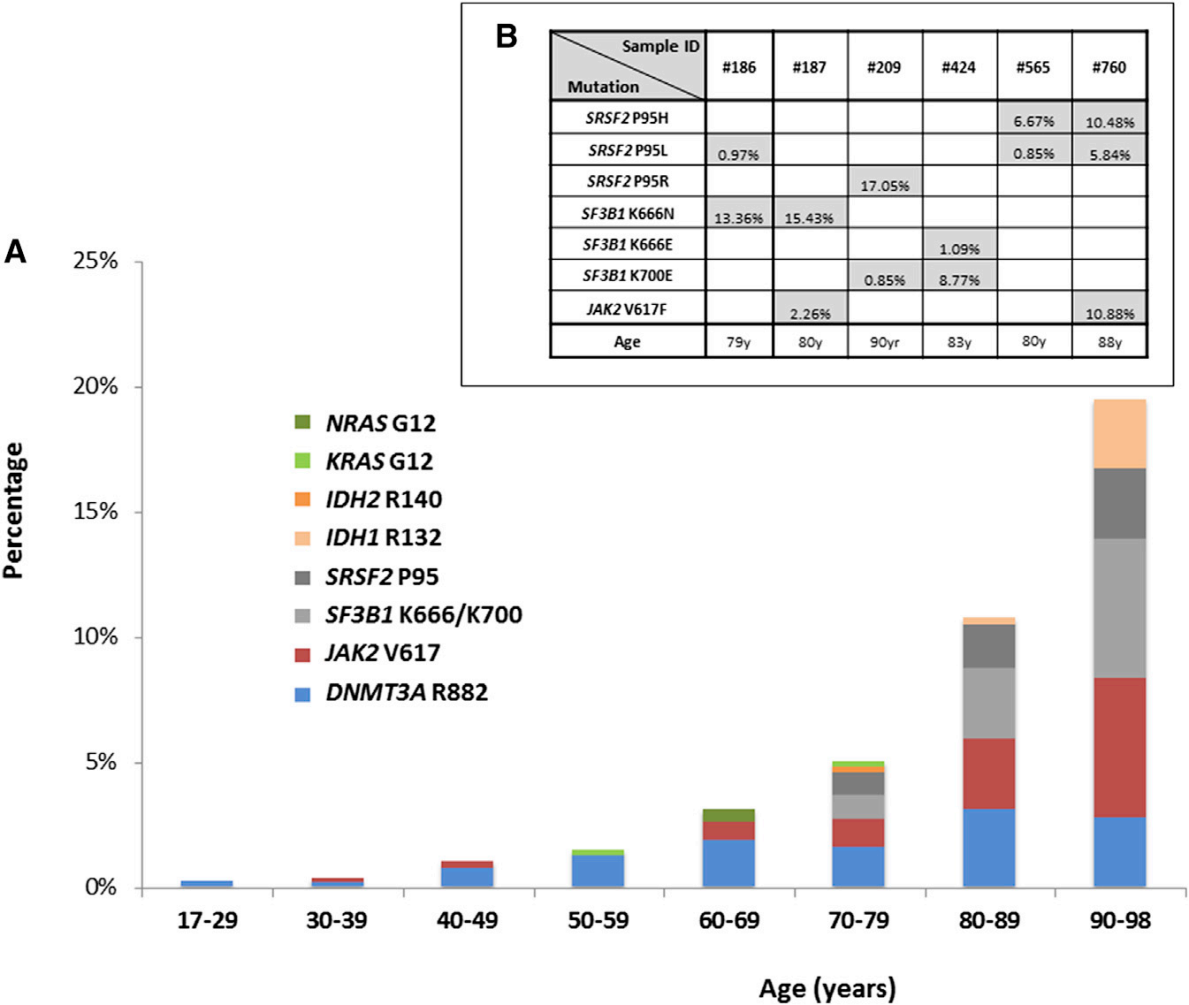
Prevalence and Age Distribution of Hot Spot Mutations Driving Clonal Hemopoiesis (A) Prevalence of mutations driving clonal hemopoiesis by age. (B) Samples with more than one mutation, variant allele fraction (VAF) of each mutation present, and age of participant. Also see Figure S1 for age distribution of all participants.

We obtained adequate coverage (≥1,000 reads at all studied hot spots) from 4,067 blood DNA samples and identified mutation-bearing clones in 105 of these. Of note, not all hot spots were studied in all samples and the derived incidence of mutations in our population as a whole was 3.24% (Table S1). However, the incidence rose significantly with age from 0.2% in the 17–29 to 19.5% in the 90–98 years age group (Figure 1A). We found one or more samples with mutations at 9 of the 15 hot spot codons studied, with VAFs varying widely within and between mutation groups (Table 2).

The most-common mutations were those affecting *DNMT3A* R882, whose incidence rose with age from 0.2% (1/489) in the 17–25 to a peak of 3.1% (11/355) in the 80–89 age group. A similar pattern was observed with *JAK2* V617F mutations (Figure 1A). By contrast, spliceosome gene mutations at *SRSF2* P95, *SF3B1* K666, and *SF3B1* K700 were exclusively observed in people aged over 70 years, rising sharply from 1.8% in those aged 70–79 to 8.3% in the 90–98 years age group. Among all samples, we identified only six individuals with more than one mutation; significantly, five of them had two independent spliceosome gene mutations of different VAFs (Figure 1B). Unfortunately, in each of three cases with two mutations at the same or nearby positions, neighboring SNPs were not informative and the variants could not be phased (see Supplemental Experimental Procedures).Occasional mutations in the genes *IDH1*, *IDH2*, *NRAS*, and *KRAS* were also seen. Except for three samples with IDH1/2 mutations, hemoglobin concentrations did not differ significantly between individuals with and without hot spot mutations (Figure S3A). For samples with full blood count results available, *JAK2* V617F mutant cases had a higher platelet count (albeit within the normal range) than “no mutation cases,” whereas other results did not differ (Figure S3B). No hot spot mutations were found in the few cord blood (n = 18) and post-transplantation (n = 32) samples studied.

Finally, despite using a very sensitive method and a mutation-calling script written specifically for this purpose, no samples with *NPM1* mutations of VAF ≥ 0.008 were identified. In fact, variant reads reporting a canonical *NPM1* mutation (mutation A; TCTG duplication) were detected in only 1 of 4,067 samples at a VAF of 0.0012 (4/3,466 reads).

## Discussion

Hematological malignancies develop through the serial acquisition of somatic mutations in a process that can take many years or even decades (Ford et al., 1998; Kyle et al., 2002). Also, it is clear that the presence of hemopoietic cells carrying leukemia-associated mutations is only followed by the onset of hematological malignancies in a minority of cases (Busque et al., 2012; Genovese et al., 2014; Jacobs et al., 2012; Jaiswal et al., 2014; Laurie et al., 2012; Xie et al., 2014). In order to understand the incidence and clonal dynamics of pre-leukemic clonal hemopoiesis, we interrogated 15 leukemia-associated mutation hot spots using a highly sensitive methodology able to detect small clones with mutations.

We show that clonal hemopoiesis is rare in the young but becomes common with advancing age. In particular, we observed that ARCH driven by the mutations studied here doubled in frequency in successive decades after the age of 50, rising from 1.5% in those aged 50–59 to 19.5% in those aged 90–98 (Figure 1). Of note, 61 of 112 clones identified had a VAF ≤ 3% (Table 2), and it is likely that most of these would not have been detected by conventional exome sequencing, which gives lower than 10-fold average coverage compared to the current study (see Table S2 for comparison to such studies), with some recurrently mutated regions giving particularly low coverage (Genovese et al., 2014). Notably, our study did not search for non-hot-spot mutations associated with ARCH such as those affecting genes *TET2* and *ASXL1* or *DNMT3A* codons other than R882 (Genovese et al., 2014; Jaiswal et al., 2014; Xie et al., 2014). Assuming that the incidence of small clones is similar for such mutations as for the hot spot mutations we studied here, the mean projected true incidence of ARCH driven by leukemia-associated mutations in those older than 90 years is greater than 70% (Figure S4). This makes clonal hemopoiesis an almost inevitable consequence of advanced aging.

Another significant finding of our study is the disparate age distribution of ARCH associated with different mutation types. In particular, we found that, although *DNMT3A* R882 and *JAK2* V617F mutations become more common with age, they were also found in younger individuals. This is in keeping with the increasing cumulative likelihood of their stochastic acquisition with the passage of time. In contrast, spliceosome gene mutations were found exclusively in those aged 70 years or older, replicating the sharp rise beyond this age in the incidence of MDSs driven by these mutations and the fact that, among unselected MDS patients, those with spliceosome mutations are significantly older than those without (Haferlach et al., 2014; Lin et al., 2014; Papaemmanuil et al., 2013; Wu et al., 2012). Exome-sequencing studies describe a much-lower rate of spliceosome mutations (Genovese et al., 2014; Jaiswal et al., 2014; Xie et al., 2014), but this is again likely to reflect their lower sensitivity for detecting small clones, which was a particular limitation at spliceosome mutation hot spots as these were captured/sequenced at lower-than-average depths (Table S2). In our study, 19/33 *SF3B1*- or *SRSF2*-associated clones had a VAF ≤ 5%, with 13 of these at VAFs ≤ 3% (Table 2), the majority of which would not have been detected by low-coverage sequencing. The identification of ARCH driven by spliceosome gene mutations is in keeping with the fact that these are founding mutations in the clonal evolution of MDS and related hematological malignancies (Cazzola et al., 2013; Haferlach et al., 2014; Papaemmanuil et al., 2013).

**Figure 2:**
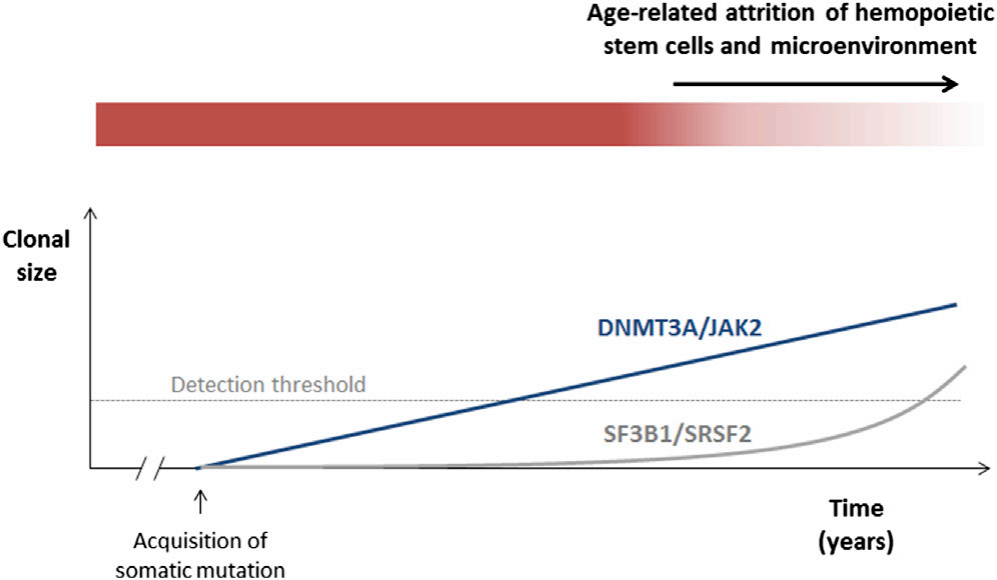
Proposed Kinetics of Hemopoietic Clones Driven by Different Gene Mutations Mutations such as *DNMT3A* R882H/C or *JAK2* V617F drive a slow but inexorable clonal expansion, leading to the outgrowth of a detectable clone after a certain latency. By contrast, mutations affecting spliceosome genes, such as *SF3B1* and *SRSF2*, and acquired at the same age for the purposes of this model give no proliferative advantage initially but do so later in the context of an aging hemopoietic compartment. Their effects may operate by prolonging stem cell survival and repopulating fitness beyond that of normal stem cells or by exploiting cell-extrinsic changes in the aging microenvironment.

We propose that the exclusive identification of spliceosome gene mutations in those aged ≥70 years can be explained by differences in the prevailing pressures on clonal selection at different ages, which can in turn explain how different gene mutations can generate detectable clonal expansions at different ages (Figure 2). The alternatives are that spliceosome mutations are associated with slower rates of clonal expansion or that they are detected later because they contribute less to circulating leukocytes. Both of these scenarios are less plausible, given the complete absence of such mutations even at low VAFs in younger age groups. For any somatic mutation imparting a clonal advantage to a stem/progenitor cell and leading to the generation of a steadily expanding clone, one would expect such a clone to be detectable at a smaller size at earlier and a larger size at later time points, as is the case for *DNMT3A* R882 and *JAK2* V617 mutations. Instead, clones (of any size) driven by mutant *SRSF2* and *SF3B1* were observed exclusively in individuals aged 70 years or older, suggesting that these only begin to expand later in life. Furthermore, considerable support for the presence of a different selection milieu comes from the observation that five of six patients with multiple mutations harbored two independent spliceosome gene mutations, indicative of convergent evolution, i.e., evolution to overcome a shared selective pressure or to exploit a shared environment (Greaves and Maley, 2012; Rossi et al., 2008).

It is tempting to consider the nature of age-related changes in normal hemopoiesis that make it permissive to the outgrowth of clones driven by spliceosome mutations. HSCs do not operate in isolation; instead, their normal survival and behavior are closely dependent on interactions with the hemopoietic microenvironment (Calvi et al., 2003; Rossi et al., 2008; Zhang et al., 2003). Therefore, both cell-intrinsic and microenvironmental factors influence hemopoietic aging (Rossi et al., 2008; Woolthuis et al., 2011). For example, there is good evidence for age-related changes in cell-intrinsic properties of HSCs in both mice (Chambers et al., 2007; Rossi et al., 2005) and humans (Rübe et al., 2011; Taraldsrud et al., 2009), and it is also clear that aging has a profound effect on the hemopoietic niche, reducing its ability to sustain polyclonal hemopoiesis, favoring oligo- or monoclonality instead (Vas et al., 2012). These and many other observations provide strong evidence that changes in the hemopoietic system subject HSCs to changing pressures during normal aging, driving clonal selection (Rossi et al., 2008).

A striking example of such selection was described in a 115-year-old woman whose peripheral white blood cells were shown to be primarily the offspring of only two related HSC clones, whose cargo of approximately 450 somatic mutations did not include known leukemogenic mutations (Holstege et al., 2014). In the absence of somatic driver mutations, it is probable that such selection is driven by well-demonstrated epigenetic differences between individual HSCs (Fraga et al., 2005) or by stochastic events. Furthermore, clonal hemopoiesis in the absence of a known leukemia-driver mutation was also well documented recently (Genovese et al., 2014), and whereas unknown or undetected drivers may be responsible for many cases of this phenomenon, it is also highly plausible that a stochastic process of clonal selection or loss may operate in others. Our study provides evidence that spliceosome gene mutations offer a means to exploit age-related changes in hemopoiesis to drive clonal hemopoiesis in advanced old age, an observation that blurs the boundary between “driver” and “passenger” mutations. Such a context dependency is not a surprising attribute for the effects of spliceosome mutations, which have not, so far, been shown to impart a primary proliferative advantage to normal hemopoietic stem and progenitor cells (Matsunawa et al., 2014; Visconte et al., 2012).

A final important finding of our study was the almost complete absence of canonical *NPM1* mutations in our collection of more than 4,000 people, despite the use of a highly sensitive assay for their detection, designed specifically for this study. Among more than 10 million mapped reads covering this mutation hot spot, we identified only four reads in a single sample reporting a canonical mutation (mutation A; TCTG duplication). Given their frequency in myeloid leukemia (Cancer Genome Atlas Research Network, 2013) and the fact that they are not late mutations (Krönke et al., 2013; Shlush et al., 2014), this observation frames *NPM1* mutations as “gatekeepers” of leukemogenesis, i.e., their acquisition appears to be closely associated with the development of frank leukemia. In this light, the frequent co-occurrence of *DNMT3A* and *NPM1* mutations suggests that the former behave as “rafts” that enable *NPM1* mutant clones to be founded and expanded, thus facilitating onward evolution toward acute myeloid leukemia.

We used a highly sensitive method to search for evidence of clonal hemopoiesis driven by 15 recurrent leukemogenic mutations in more than 4,000 individuals. Our results demonstrate that the incidence of clonal hemopoiesis is much higher than suggested by exome-sequencing studies, that spliceosome gene mutations drive clonal outgrowth primarily in the context of an aging hemopoietic compartment, and that *NPM1* mutations do not drive ARCH, indicating that their acquisition is closely associated with frank leukemia.

## Experimental Procedures

### Patient Samples

Samples were obtained with written informed consent and in accordance with the Declaration of Helsinki and appropriate ethics committee approvals from all participants (approval reference numbers 10/H0604/02, 07/MRE05/44, and 05/Q0106/74). Maternal consent was obtained for the use of cord blood samples. Samples were obtained from 3,067 blood donors aged 17–70 years (WTCCC; UK Blood Services 1 [UKBS1] and UKBS2 common controls), 1,152 unselected individuals aged 60–98 years (UKHLS; https://www.understandingsociety.ac.uk/), 32 patients that had undergone a hemopoietic stem cell transplant (12 autologous and 20 allogeneic; Tables S3 and S4) 1 month to 14 years previously, and 18 cord blood samples. Age distribution of the WTCCC and UKHLS cohorts/samples is shown in Figure S1. Hemoglobin concentrations were available for a total of 3,587 of the 4,067 samples from which adequate sequencing data were obtained for analysis, including 102 of 105 samples with mutations. Full blood count results were available for 2,952 WTCCC samples. The average blood donation frequency for WTCCC donors was 1.6 donations of one unit per year. Details of donations by individual participants were not available.

### Targeted Sequencing

Genomic DNA was used to simultaneously amplify several gene loci using multiplex PCR, in order to capture and analyze 15 mutational hot spots enriched for, but not exclusive to, targets of mutations thought to arise early in leukemogenesis (Table 1). We used three multiplex primer combinations (Plex1-3), guided by our findings, to capture the targeted mutational hot spots (Table S1). Primers were designed using the Hi-Plex PCR-MPS (massively parallel sequencing) strategy (Nguyen-Dumont et al., 2013), except for *JAK2* V617 and “Plex2” primers, which were designed using MPRIMER (Shen et al., 2010). These and additional primer sequences used in each Plex and details of PCR- and DNA-sequencing protocols are detailed in Supplemental Experimental Procedures. Methodological validation experiments are shown in Figure S2.

### Bioinformatic Analysis

Sequencing data were aligned to the human reference genome (hg19) using BWA. Subsequently, the SAMTOOLS pileup command was used to generate pileup files from the generated bam files (version 0.1.8; http://samtools.sourceforge.net; Li et al., 2009). A flexible in-house Perl script generated by our group, MIDAS (Conte et al., 2013), was modified in order to interrogate only the hot spot nucleotide positions of interest (those with reported mutations in the COSMIC database; Forbes et al., 2015) on the pileup file, considering only those reads with a sequence quality higher than 25 and a mapping quality higher than 15. For each sample, the numbers of reads reporting the reference and variant alleles at each position were extracted. VAFs were derived by dividing the number of reads reporting the most-frequent variant nucleotide to the total. In order to detect NPM1 mutations with high sensitivity, we wrote a bespoke Perl script described in Supplemental Experimental Procedures.

### Statistical Analyses and Mutation-Calling Threshold

We chose a threshold VAF of ≥0.008 (0.8%) to “call” clones with a heterozygous mutation representing ≥1.6% of blood leukocytes. From validation experiments and data analysis (see Supplemental Experimental Procedures and Figure S2D), we determined that the maximum false-positive error rate for calling a mutation (VAF ≥ 0.008) due to variant allele counts that are solely due to PCR-MiSeq error was negligible (p < 10^−5^). For comparisons of blood cell counts and hemoglobin concentrations, we used non-paired t tests. For summary statistics of read coverage (Table S2) and for the purposes of deriving an estimate of the overall incidence of clonal hemopoiesis (Figure S4), we used published tables of all mutations reported by three recent studies that employed whole-exome-sequencing analyses to identify individuals with clonal hemopoiesis (Genovese et al., 2014; Jaiswal et al., 2014; Xie et al., 2014).

## Author Contributions

G.S.V. conceived and designed the study. G.S.V. and T. McKerrell supervised the study, analyzed data, and wrote the manuscript. N.P. and T. McKerrell performed experimental procedures. I.V. and T. Moreno wrote scripts and performed bioinformatics analysis. H.P., T. McKerrell, and G.S.V. performed statistical analyses. E.Z., C.S.G., M.A.Q., and R.R. contributed to study strategy and to technical and analytical aspects. U.S.S.G., E.Z., W.O, J.C., C.C., J.B., J.S., C.H., M.A.S., and D.R.F. contributed to sample acquisition and subject recruitment.

## Figures and Tables

**Table 1:** Mutation Hot Spots Interrogated in This Study

Gene	Target Codon
DNMT3A	R882
JAK2	V617
NPM1	L287
SRSF2	P95
SF3B1	K666
SF3B1	K700
IDH1	R132
IDH2	R140
IDH2	R172
KRAS	G12
NRAS	G12
NRAS	Q61
KIT	D816
FLT3	D835
FLT3	N676

Also see Table S1 for detailed information about numbers of samples screened for each mutation.

**Table 2:** Amino Acid Consequences and VAFs of the 112 Clonal Mutations Identified in This Study

Mutation Hot Spot	Codon	VAF (%)	Age	Mutation Hot Spot	Codon	VAF (%)	Age	Mutation Hot Spot	Codon	VAF (%)	Age
*DNMT3A* R882	p.R882H	4.14	25		p.R882H	32.02	81	*IDH1 R132*	p.R132H	42.13	84
	p.R882C	2.33	35		p.R882H	1.14	81		p.R132C	0.92	92
	p.R882H	3.80	42		p.R882H	3.06	81	*IDH2 R140*	p.R140Q	6.67	76
	p.R882H	4.00	42		p.R882H	2.17	81	*SRSF2 P95*	p.P95R	4.46	70
	p.R882H	1.25	43		p.R882H	1.13	82		p.P95L	3.35	72
	p.R882H	19.00	48		p.R882H	1.46	82		p.P95H	0.86	73
	p.R882H	1.18	49		p.R882C	2.62	82		p.P95H	0.84	77
	p.R882S	1.74	49		p.R882C	6.15	89		p.P95L	0.97	79†
	p.R882H	9.87	50		p.R882C	2.00	94		p.P95L	0.85	80††
	p.R882H	0.83	51	*JAK2V617F*	p.V617F	1.56	34		p.P95H	6.67	80††
	p.R882C	1.10	51		p.V617F	4.91	42		p.P95L	0.96	81
	p.R882C	12.50	52		p.V617F	7.72	45		p.P95H	6.40	82
	p.R882C	1.28	53		p.V617F	0.85	62		p.P95L	2.74	85
	p.R882C	2.47	54		p.V617F	25.44	64		p.P95R	7.52	87
	p.R882H	1.95	55		p.V617F	7.41	65		p.P95L	5.84	88^∗∗^
	p.R882C	30.22	55		p.V617F	1.03	67		p.P95H	10.48	88^∗∗^
	p.R882C	1.22	56		p.V617F	0.88	71		p.P95R	2.71	88
	p.R882H	0.91	58		p.V617F	3.75	71		p.P95R	17.05	90‡
	p.R882H	4.17	60		p.V617F	1.16	75	*SF3B1* K700	p.K700E	1.04	76
	p.R882H	5.90	60		p.V617F	2.30	77		p.K700E	6.63	81
	p.R882H	9.60	60		p.V617F	1.92	78		p.K700E	0.79	82
	p.R882H	2.73	60		p.V617F	2.26	80^∗^		p.K700E	12.59	83
	p.R882C	9.33	60		p.V617F	4.25	80		p.K700E	8.77	83‡‡
	p.R882H	7.03	61		p.V617F	1.92	80		p.K700E	1.02	84
	p.R882C	1.21	61		p.V617F	3.71	80		p.K700E	0.85	90‡
	p.R882H	0.86	63		p.V617F	15.48	81		p.K700E	1.37	90
	p.R882H	2.54	64		p.V617F	1.21	82	*SF3B1* K666	p.K666N	1.33	70
	p.R882H	3.19	67		p.V617F	1.62	85		p.K666N	5.01	79
	p.R882H	2.74	70		p.V617F	0.83	85		p.K666N	13.36	79†
	p.R882H	4.27	74		p.V617F	1.98	86		p.K666N	15.43	80^∗^
	p.R882H	0.85	74		p.V617F	25.94	88		p.K666N	4.60	81
	p.R882H	0.85	75		p.V617F	10.88	88^∗∗^		p.K666E	1.09	83‡‡
	p.R882C	1.12	77		p.V617F	2.94	90		p.K666N	35.11	86
	p.R882C	1.15	78		p.V617F	1.23	90		p.K666N	19.70	86
	p.R882H	1.26	79	*KRAS G12*	p.G12 R	0.94	55		p.K666N	16.55	86
	p.R882H	16.66	80		p.G12S	2.78	78		p.K666E	3.34	95
	p.R882C	4.28	80	*NRAS G12*	p.G12S	1.50	61				
	p.R882C	3.66	80		p.G12D	0.96	62				

Mutations identified in the same sample are highlighted with the same symbol (^∗^, ^∗∗^, †, ††, ‡, and ‡‡).

## Accession Numbers

The European Genome-Phenome Archive (EGA) accession number for the sequencing data reported in this paper is EGAS00001000814.

## Supplemental Information

Document S1. Supplemental Experimental Procedures, Figures S1–S4, and Tables S1–S4

Document S2. Article plus Supplemental Information

## References

[bib1] Busque L., Patel J.P., Figueroa M.E., Vasanthakumar A., Provost S., Hamilou Z., Mollica L., Li J., Viale A., Heguy A. (2012). Recurrent somatic TET2 mutations in normal elderly individuals with clonal hematopoiesis. Nat. Genet..

[bib2] Calvi L.M., Adams G.B., Weibrecht K.W., Weber J.M., Olson D.P., Knight M.C., Martin R.P., Schipani E., Divieti P., Bringhurst F.R. (2003). Osteoblastic cells regulate the haematopoietic stem cell niche. Nature.

[bib3] Cancer Genome Atlas Research Network (2013). Genomic and epigenomic landscapes of adult de novo acute myeloid leukemia. N. Engl. J. Med..

[bib4] Cazzola M., Della Porta M.G., Malcovati L. (2013). The genetic basis of myelodysplasia and its clinical relevance. Blood.

[bib5] Chambers S.M., Shaw C.A., Gatza C., Fisk C.J., Donehower L.A., Goodell M.A. (2007). Aging hematopoietic stem cells decline in function and exhibit epigenetic dysregulation. PLoS Biol..

[bib6] Conte N., Varela I., Grove C., Manes N., Yusa K., Moreno T., Segonds-Pichon A., Bench A., Gudgin E., Herman B. (2013). Detailed molecular characterisation of acute myeloid leukaemia with a normal karyotype using targeted DNA capture. Leukemia.

[bib7] Ding L., Ley T.J., Larson D.E., Miller C.A., Koboldt D.C., Welch J.S., Ritchey J.K., Young M.A., Lamprecht T., McLellan M.D. (2012). Clonal evolution in relapsed acute myeloid leukaemia revealed by whole-genome sequencing. Nature.

[bib8] Forbes S.A., Beare D., Gunasekaran P., Leung K., Bindal N., Boutselakis H., Ding M., Bamford S., Cole C., Ward S. (2015). COSMIC: exploring the world’s knowledge of somatic mutations in human cancer. Nucleic Acids Res..

[bib9] Ford A.M., Bennett C.A., Price C.M., Bruin M.C., Van Wering E.R., Greaves M. (1998). Fetal origins of the TEL-AML1 fusion gene in identical twins with leukemia. Proc. Natl. Acad. Sci. USA.

[bib10] Fraga M.F., Ballestar E., Paz M.F., Ropero S., Setien F., Ballestar M.L., Heine-Suñer D., Cigudosa J.C., Urioste M., Benitez J. (2005). Epigenetic differences arise during the lifetime of monozygotic twins. Proc. Natl. Acad. Sci. USA.

[bib11] Genovese G., Kähler A.K., Handsaker R.E., Lindberg J., Rose S.A., Bakhoum S.F., Chambert K., Mick E., Neale B.M., Fromer M. (2014). Clonal hematopoiesis and blood-cancer risk inferred from blood DNA sequence. N. Engl. J. Med..

[bib12] Greaves M., Maley C.C. (2012). Clonal evolution in cancer. Nature.

[bib13] Haferlach T., Nagata Y., Grossmann V., Okuno Y., Bacher U., Nagae G., Schnittger S., Sanada M., Kon A., Alpermann T. (2014). Landscape of genetic lesions in 944 patients with myelodysplastic syndromes. Leukemia.

[bib14] Holstege H., Pfeiffer W., Sie D., Hulsman M., Nicholas T.J., Lee C.C., Ross T., Lin J., Miller M.A., Ylstra B. (2014). Somatic mutations found in the healthy blood compartment of a 115-yr-old woman demonstrate oligoclonal hematopoiesis. Genome Res..

[bib15] Jacobs K.B., Yeager M., Zhou W., Wacholder S., Wang Z., Rodriguez-Santiago B., Hutchinson A., Deng X., Liu C., Horner M.J. (2012). Detectable clonal mosaicism and its relationship to aging and cancer. Nat. Genet..

[bib16] Jaiswal S., Fontanillas P., Flannick J., Manning A., Grauman P.V., Mar B.G., Lindsley R.C., Mermel C.H., Burtt N., Chavez A. (2014). Age-related clonal hematopoiesis associated with adverse outcomes. N. Engl. J. Med..

[bib17] Krönke J., Bullinger L., Teleanu V., Tschürtz F., Gaidzik V.I., Kühn M.W., Rücker F.G., Holzmann K., Paschka P., Kapp-Schwörer S. (2013). Clonal evolution in relapsed NPM1-mutated acute myeloid leukemia. Blood.

[bib18] Kyle R.A., Therneau T.M., Rajkumar S.V., Offord J.R., Larson D.R., Plevak M.F., Melton L.J. (2002). A long-term study of prognosis in monoclonal gammopathy of undetermined significance. N. Engl. J. Med..

[bib19] Laurie C.C., Laurie C.A., Rice K., Doheny K.F., Zelnick L.R., McHugh C.P., Ling H., Hetrick K.N., Pugh E.W., Amos C. (2012). Detectable clonal mosaicism from birth to old age and its relationship to cancer. Nat. Genet..

[bib20] Li H., Handsaker B., Wysoker A., Fennell T., Ruan J., Homer N., Marth G., Abecasis G., Durbin R., 1000 Genome Project Data Processing Subgroup (2009). The Sequence Alignment/Map format and SAMtools. Bioinformatics.

[bib21] Lin C.C., Hou H.A., Chou W.C., Kuo Y.Y., Wu S.J., Liu C.Y., Chen C.Y., Tseng M.H., Huang C.F., Lee F.Y. (2014). SF3B1 mutations in patients with myelodysplastic syndromes: the mutation is stable during disease evolution. Am. J. Hematol..

[bib22] Matsunawa M., Yamamoto R., Sanada M., Sato-Otsubo A., Shiozawa Y., Yoshida K., Otsu M., Shiraishi Y., Miyano S., Isono K. (2014). Haploinsufficiency of Sf3b1 leads to compromised stem cell function but not to myelodysplasia. Leukemia.

[bib23] Nguyen-Dumont T., Pope B.J., Hammet F., Southey M.C., Park D.J. (2013). A high-plex PCR approach for massively parallel sequencing. Biotechniques.

[bib24] Nowell P.C. (1976). The clonal evolution of tumor cell populations. Science.

[bib25] Papaemmanuil E., Gerstung M., Malcovati L., Tauro S., Gundem G., Van Loo P., Yoon C.J., Ellis P., Wedge D.C., Pellagatti A., Chronic Myeloid Disorders Working Group of the International Cancer Genome Consortium (2013). Clinical and biological implications of driver mutations in myelodysplastic syndromes. Blood.

[bib26] Rossi D.J., Bryder D., Zahn J.M., Ahlenius H., Sonu R., Wagers A.J., Weissman I.L. (2005). Cell intrinsic alterations underlie hematopoietic stem cell aging. Proc. Natl. Acad. Sci. USA.

[bib27] Rossi D.J., Jamieson C.H., Weissman I.L. (2008). Stems cells and the pathways to aging and cancer. Cell.

[bib28] Rübe C.E., Fricke A., Widmann T.A., Fürst T., Madry H., Pfreundschuh M., Rübe C. (2011). Accumulation of DNA damage in hematopoietic stem and progenitor cells during human aging. PLoS ONE.

[bib29] Shen Z., Qu W., Wang W., Lu Y., Wu Y., Li Z., Hang X., Wang X., Zhao D., Zhang C. (2010). MPprimer: a program for reliable multiplex PCR primer design. BMC Bioinformatics.

[bib30] Shlush L.I., Zandi S., Mitchell A., Chen W.C., Brandwein J.M., Gupta V., Kennedy J.A., Schimmer A.D., Schuh A.C., Yee K.W., HALT Pan-Leukemia Gene Panel Consortium (2014). Identification of pre-leukaemic haematopoietic stem cells in acute leukaemia. Nature.

[bib31] Taraldsrud E., Grøgaard H.K., Solheim S., Lunde K., Fløisand Y., Arnesen H., Seljeflot I., Egeland T. (2009). Age and stress related phenotypical changes in bone marrow CD34+ cells. Scand. J. Clin. Lab. Invest..

[bib32] Vas V., Senger K., Dörr K., Niebel A., Geiger H. (2012). Aging of the microenvironment influences clonality in hematopoiesis. PLoS ONE.

[bib33] Visconte V., Rogers H.J., Singh J., Barnard J., Bupathi M., Traina F., McMahon J., Makishima H., Szpurka H., Jankowska A. (2012). SF3B1 haploinsufficiency leads to formation of ring sideroblasts in myelodysplastic syndromes. Blood.

[bib34] Woolthuis C.M., de Haan G., Huls G. (2011). Aging of hematopoietic stem cells: Intrinsic changes or micro-environmental effects?. Curr. Opin. Immunol..

[bib35] Wu S.J., Kuo Y.Y., Hou H.A., Li L.Y., Tseng M.H., Huang C.F., Lee F.Y., Liu M.C., Liu C.W., Lin C.T. (2012). The clinical implication of SRSF2 mutation in patients with myelodysplastic syndrome and its stability during disease evolution. Blood.

[bib36] Xie M., Lu C., Wang J., McLellan M.D., Johnson K.J., Wendl M.C., McMichael J.F., Schmidt H.K., Yellapantula V., Miller C.A. (2014). Age-related mutations associated with clonal hematopoietic expansion and malignancies. Nat. Med..

[bib37] Zhang J., Niu C., Ye L., Huang H., He X., Tong W.G., Ross J., Haug J., Johnson T., Feng J.Q. (2003). Identification of the haematopoietic stem cell niche and control of the niche size. Nature.

