# Supplementary material for: Leukemia-Associated Somatic Mutations Drive Distinct Patterns of Age-Related Clonal Hemopoiesis
Source: Cell Rep. 2015 Feb 26;10(8):1239–45. doi: 10.1016/j.celrep.2015.02.005 (PMC4542313; doi:10.1016/j.celrep.2015.02.005)
Supplement: Document S1. Supplemental Experimental Procedures, Figures S1–S4, and Tables S1–S4 [file mmc1.pdf]

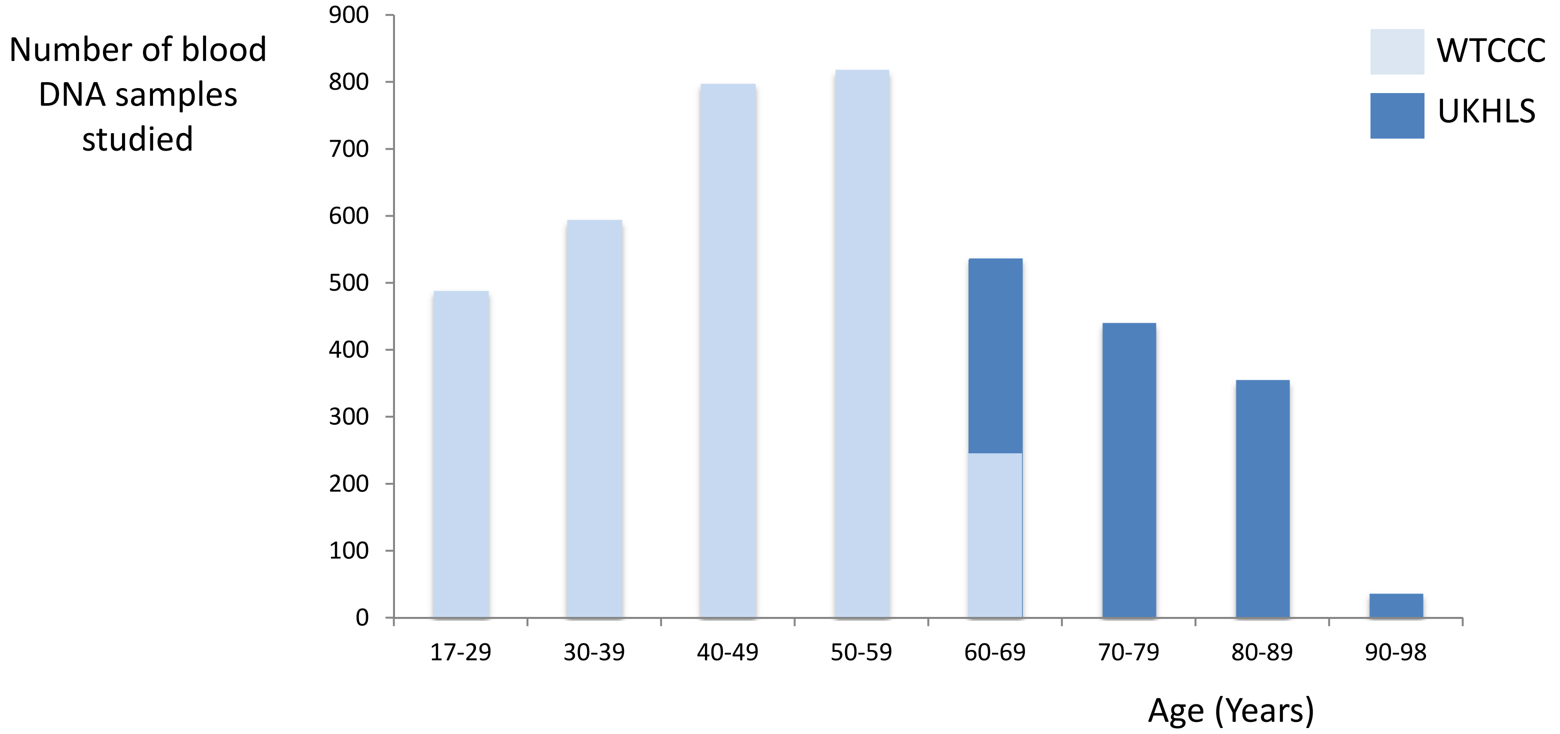
Number of blood DNA samples studied
900
800
700
600
500
400
300
200
100
0
17-29
30-39
40-49
50-59
60-69
70-79
80-89
90-98
Age (Years)
WTCCC
UKHLS

Figure S1

A B C D

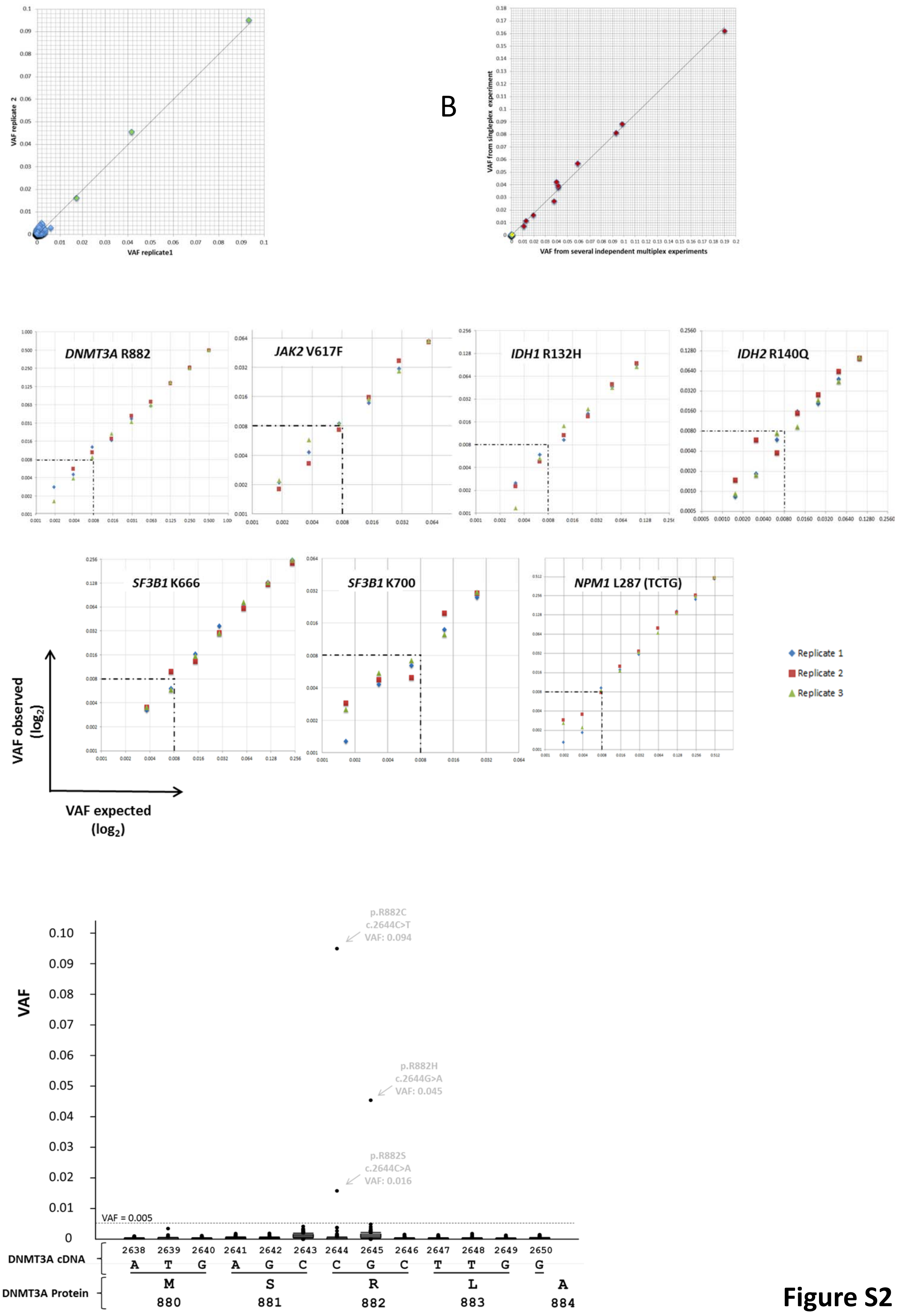

Figure S2

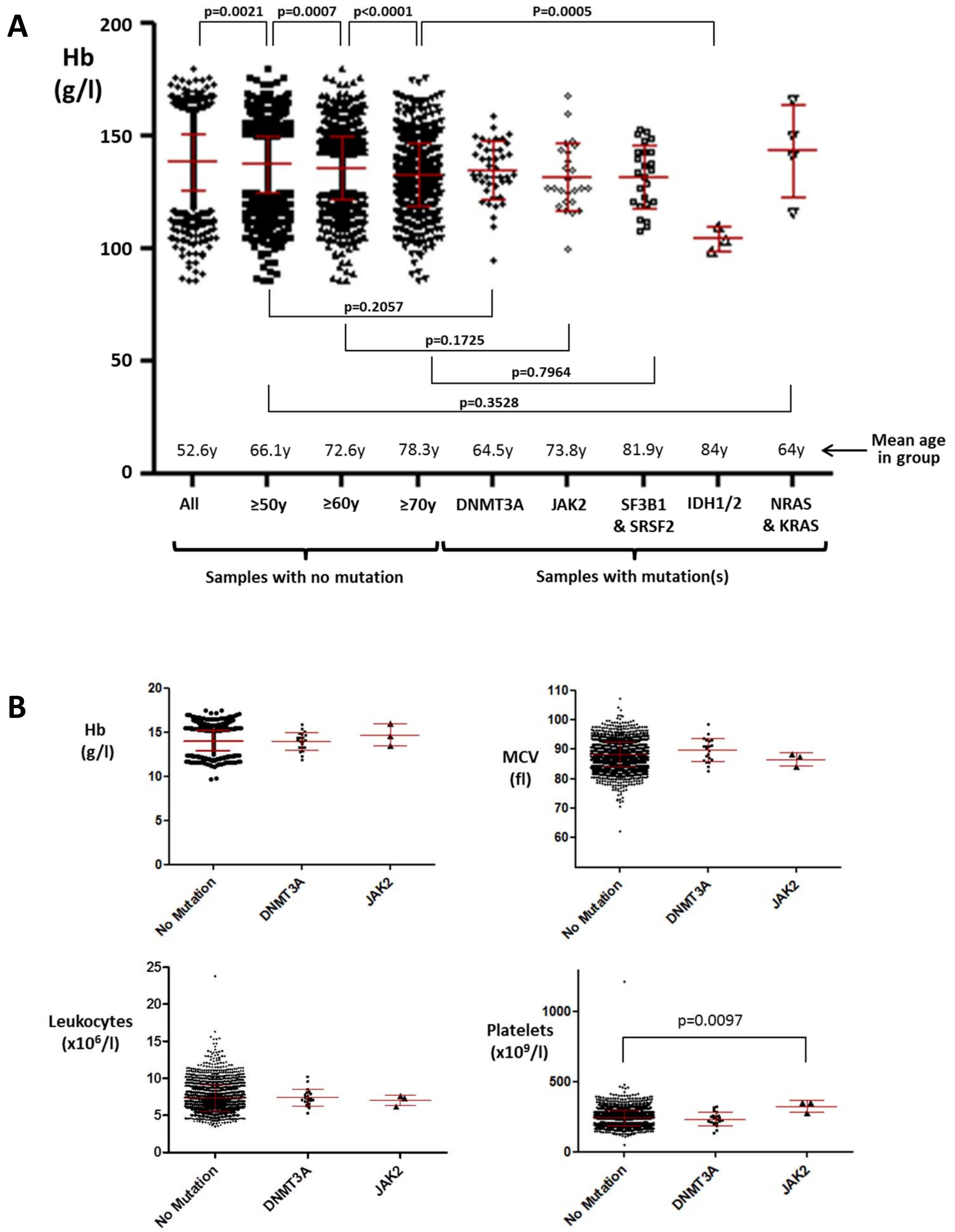
A
Hb
(g/l)
p=0.0021
p=0.0007
p<0.0001
P=0.0005
p=0.2057
p=0.1725
p=0.7964
p=0.3528
52.6y
66.1y
72.6y
78.3y
64.5y
73.8y
81.9y
84y
64y
Mean age in group
All
≥50y
≥60y
≥70y
DNMT3A
JAK2
SF3B1 & SRSF2
IDH1/2
NRAS & KRAS
Samples with no mutation
Samples with mutation(s)
B
Hb (g/l)
MCV (fl)
Leukocytes (x10⁶/l)
Platelets (x10⁹/l)
p=0.0097
No Mutation
DNMT3A
JAK2

Figure S3

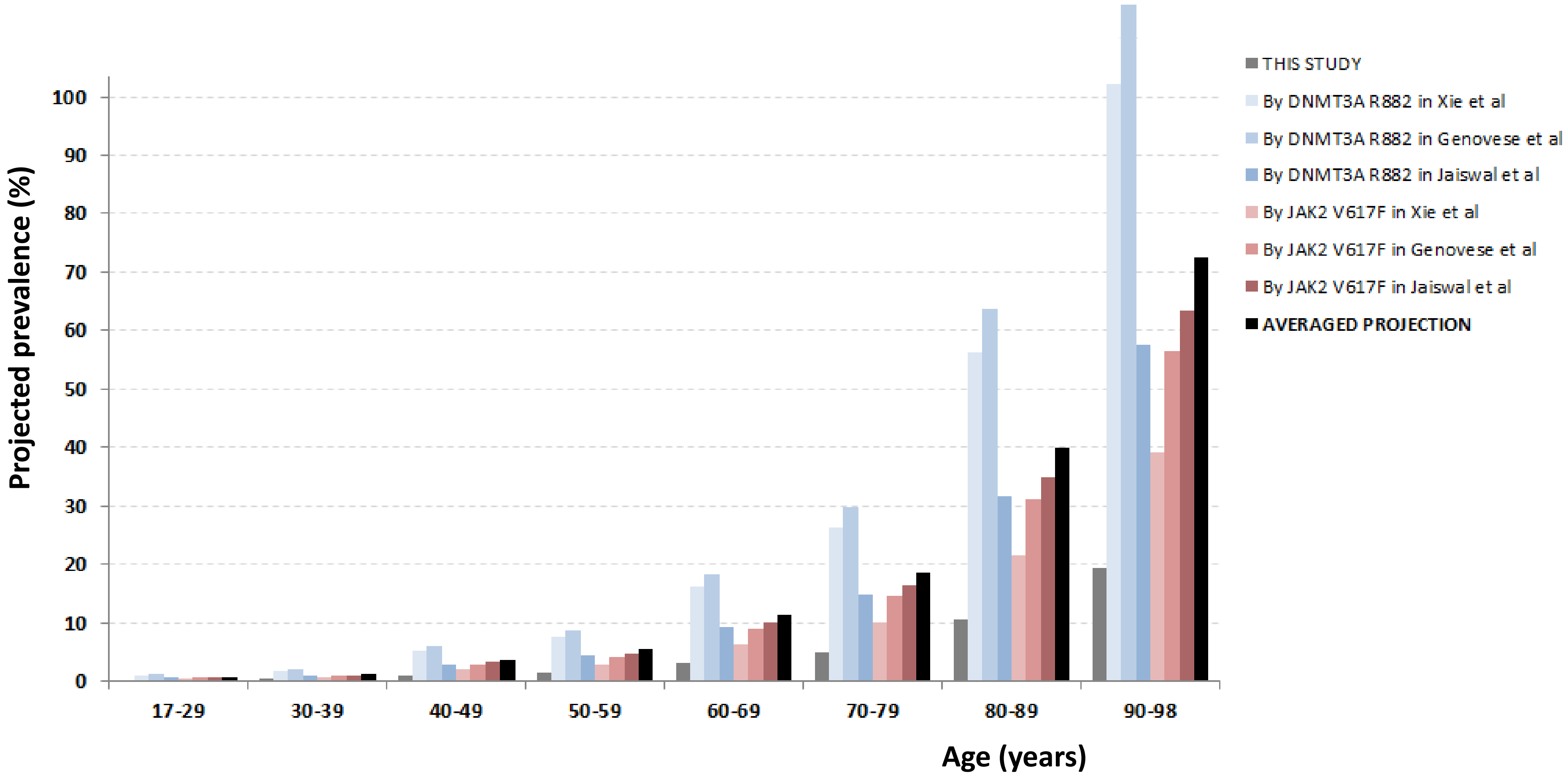

Projected prevalence (%)
100
90
80
70
60
50
40
30
20
10
0
17-29
30-39
40-49
50-59
60-69
70-79
80-89
90-98
Age (years)
THIS STUDY
By DNMT3A R882 in Xie et al
By DNMT3A R882 in Genovese et al
By DNMT3A R882 in Jaiswal et al
By JAK2 V617F in Xie et al
By JAK2 V617F in Genovese et al
By JAK2 V617F in Jaiswal et al
AVERAGED PROJECTION

Figure S4

SUPPLEMENTAL FIGURE LEGENDS

Figure S1. Numbers of individuals/DNA samples studied for the presence of clonal hemopoiesis

The sample group from which blood DNA samples were obtained is indicated. Samples were studied with different but overlapping sets of multiplex PCR primers (Plex 1-3, see Table S1). WTCC= Wellcome Trust Case Control Consortium, UKHLS= United Kingdom Household Longitudinal Study.

Related to experimental procedures

Figure S2. Methodological validation of multiplex PCR-MiSeq

A. To validate the reproducibility, accuracy and error rate of our multiplex PCR-MiSeq sequencing protocol, we studied the same 384 blood DNA samples twice in independent experiments and derived VAF values for the two *DNMT3A* R882 mutation hotspot positions, c.2644 and c.2645. The 371 samples with more than 1000 reads in both experiments are plotted (i.e. 2 replicates of 742 VAF datapoints). VAFs from the same sample and position from experiment 1 are plotted against the equivalent value from experiment 2. The 739 samples with VAFs ≤0.006 (blue diamonds) show relatively poor correlation in keeping with PCR/sequencing error at these very low VAF values. Nevertheless, no VAF values >0.006 are seen in either replicate except for the three real mutant samples (green diamonds) whose higher VAFs correlate extremely well**.**

B. To validate the reproducibility of our protocol for quantifying real mutations with VAFs ≥0.008, we re-analyzed 11 samples carrying subclonal DNA mutations with VAFs from 0.01 to 0.19 (i.e. 1-19% mutant reads) and 14 samples without evidence of subclonal mutations determined in several independent multiplex PCR experiments/batches , using an independent singleplex PCR for *DNMT3A* R882 (different primers). As shown, there was excellent correlation between multiplex and singleplex VAFs for all 11 mutant samples (red diamonds). The 14 samples without evidence of subclonal mutations according to the multiplex PCR, again gave very low VAFs (<0.0004) with singleplex analysis (yellow diamonds), therefore demonstrating the robust VAF quantitation achieved using our approach.

C. To validate the linearity of VAF quantitation of our protocol for the detection of low level subclonal mutations, we analyzed serial 2-fold dilutions of primary leukaemic or cell line (OCI-AML3) DNA into unmutated cord blood DNA for the following codons: *DNMT3A* R882, *JAK2* V617F, *IDH1* R132, *IDH2* R140, *SF3B1* K666, *SF3B1* K700 and *NPM1* L287. The “expected” VAF values were derived using the VAF obtained for the neat DNA of primary leukaemic or OCI-AML3 DNA in each experiment. Results of 3 independent replicate experiments using the same diluted DNA samples are shown. All hotspot loci studied show very good correlation between replicates for VAFs ≥0.008 (0.8%), our cut-off for “calling” mutant clones.

D. To derive the likelihood of obtaining a false positive mutation call we calculated the VAF for nucleotide positions surrounding the *DNMT3A* R882 codon outside of the mutation hotspots (c.2644 and c.2645) in 371 samples. Across all samples analyzed in this way, nucleotide positions outside the mutation hotspots gave VAFs lower than 0.005 (maximum VAF was 0.0041 at position c.2643). At the two hotspot positions we observed 3 samples with VAFs much higher than 0.005 (arrows). Near identical results were obtained when the same samples were analyzed for a second time using independent PCR amplifications and sequencing. These data were used to derive the likelihood of obtaining an erroneous VAF value greater than 0.008 (i.e. a false positive mutation call).

Related to experimental procedures

Figure S3. Comparisons of blood results between participants with different mutations

A. Hemoglobin concentrations (Hb) in different age and mutation groups for all participants (WTCCC & UKHLS). Individual values are plotted and red bars represent mean +/ - standard deviation for each group. Paired t - tests were performed for the indicated comparisons and, amongst individuals with no mutations, values were found to differ significantly between age groups (p values for each comparison indicated). Therefore, for each mutation group the "no mutation" group with the most similar mean age was used as control. Only IDH1/2 mutant samples had significantly lower Hb compared to age-matched controls. The 6 samples with more than one mutation were classified according to the mutation with the highest VAF.

B. Blood count results in different WTCCC participant groups. Individual values are plotted and red bars represent mean +/- standard deviation for each group. Paired t-tests were performed comparing "No mutation" samples with the DNMT3A mutant group (n=24) and, separately, with the JAK2 mutant group (n=3). The only significant comparison ($p<0.05$) was for platelet counts, which were higher in the JAK2 group. However, values for all 3 JAK2 mutant samples were within the reference range. The one KRAS and one NRAS mutant samples identified in the WTCCC sample group were not included in these comparisons. MCV=mean corpuscular volume (of erythrocytes).

Related to Figure 1

Figure S4. Projected overall prevalence of Age-Related Clonal Haemopoiesis driven by leukemia-associated mutations

Our methodology for detecting hotspot mutations was much more sensitive than approaches used by others to date. In order to derive approximate estimates of the overall prevalence of ARCH driven by leukemia-associated mutations, we projected our findings onto those of published datasets from three recent studies that used whole-exome sequencing of blood DNA to identify individuals with ARCH (Xi et al, 2014; Genovese et al; Jaiswal et al). As the age-distribution of participants varied significantly between studies and details of age-distribution of individuals mutations were not given by the two largest studies (Genovese et al; Jaiswal et al), we used the fraction of all mutations represented by DNMT3A R882 and by JAK2 V617F in each study to derive estimates of the overall prevalence of ARCH at the sensitivity of our study (i.e. VAF≥0.008). We chose these two mutations as they are the two commonest recurrent events in our study and also because they were identified in most age groups, albeit at varying frequencies. A notable limitation of this approach is the fact that exome sequencing detects different mutations with different sensitivities. In fact sequence coverage for DNMT3A R882 was lower, whilst that for JAK2 V617 was higher than average. Also each study set a different minimum VAF for "calling" mutations (Xie et al, VAF≥0.1; Genovese et al, VAF≥0.05 and Jaiswal et al, VAF≥0.03). Nevertheless, even the most conservative of our projections indicate that ARCH is much commoner than previously considered and is likely to occur in the majority of people aged over 90 years.

Related to Figure 1

Gene	Target codon	Plex 1	Plex 2	Plex 3	Numbers studied at each locus	Number of mutations per locus	Incidence of mutations per locus (%)
DNMT3A	R882	✓	✓	✓	4067	47	1.16
JAK2	V617	✓	✓	✓	4067	25	0.61
NPM1	L287	✓	✓	✓	4067	0	0.00
SRSF2	P95	✓		✓	2577	13	0.50
SF3B1	K666	✓		✓	2577	10	0.39
SF3B1	K700	✓		✓	2577	8	0.31
IDH1	R132	✓		✓	2577	2	0.08
IDH2	R140	✓		✓	2577	1	0.04
IDH2	R172	✓		✓	2577	0	0.00
KRAS	G12		✓	✓	2606	2	0.08
NRAS	G12		✓	✓	2606	2	0.08
NRAS	Q61		✓	✓	2606	0	0.00
KIT	D816		✓	✓	2606	0	0.00
FLT3	D835		✓	✓	2606	0	0.00
FLT3	N676		✓		1490	0	0.00
Number of individuals screened using each Plex design		1531	1536	1152			
Number of individuals with adequate coverage for analysis*		1461	1490	1116			

Table S1

Study	Mutation Group	Mutation number	Read depth (coverage)*			
			Mean	SD**	Minimum	Maximum
Genovese et al, 2014	All mutations	327	91.7	51.0	11	371
	DNMT3A (all)	190	91.2	49.2	21	255
	DNMT3A R882	123	58.6	17.2	30	101
	JAK2 V617F	24	111.0	27.1	79	191
	SF3B1 K666	3	162.0	89.3	72	234
	SF3B1 K700	9	89.3	32.7	59	164
	SRSF2P95	5	59.0	10.3	47	69
Jaiswal et al, 2014	All mutations	805	94.0	53.9	16	432
	DNMT3A (all)	403	92.6	50.7	18	384
	DNMT3A R882	67	61.9	15.9	34	95
	JAK2 V617F	31	121.8	38.8	81	265
	SF3B1 K666	11	69.5	15.7	52	107
	SF3B1K700	12	82.5	26.7	38	133
	SRSF2 P95	10	51.8	11.3	35	72
Xie et al, 2014	All mutations	77	107.8	69.0	22	387
	DNMT3A (all)	18	109.3	86.8	28	387
	DNMT3A R882	6	65.5	31.6	28	115
	JAK2 V617	8	156.0	66.0	63	237
	SF3B1K666	0	n/a	n/a	n/a	n/a
	SF3B1K700	2	90.0	15.6	79	101
	SRSF2 P95	0	n/a	n/a	n/a	n/a

Table S2

Patient age	Gender	Indication for autologous HSCT	Time since HSCT (months)
61	M	Myeloma	12
68	M	Hodgkins Lymphoma	4.5
63	M	Mantle Cell Lymphoma	1
27	M	Hodgkins Lymphoma	17
53	F	Follicular Lymphoma	8
41	F	Diffuse Large B Cell Lymphoma	12
66	F	Mantle cell Lymphoma	24
63	M	Myeloma	34
57	F	Diffuse Large B Cell Lymphoma	15
55	M	Myeloma	26
51	M	Myeloma	65
49	M	NK T cell Lymphoma	15

Table S3

Patient age	Gender	Indication for Allogeneic HSCT	Time since HSCT (months)	Donor age at sampling	Donor Gender	Donor Chimerism (%)
63	M	Diffuse Large B Cell Lymphoma	5	67	F	95
52	F	Chronic Lymphocytic Leukemia	36	Unknown	M	99
52	M	Angioimmunoblastic lymphoma	18	47	F	100
61	M	Myelodysplastic syndrome (RAEB)	63	27	M	100
33	M	Non-Hodgkin's Lymphoma	94	37	F	Unknown
58	M	Acute Myeloid Leukemia	15	Unknown	M	100
59	M	Acute Myeloid Leukemia	25	Unknown	M	100
44	F	Acute Myeloid Leukemia	34	Unknown	F	100
41	M	Myeloma	22	Unknown	M	100
49	M	Acute Myeloid Leukemia	105	Unknown	M	Unknown
56	F	Acute Myeloid Leukemia	63	42	M	96
47	F	Chronic Myeloid Leukemia	170	Unknown	Unknown	Unknown
50	M	Blast crisis of Chronic Myeloid Leukemia	30	Unknown	M	Unknown
19	M	Aplastic Anaemia	13	15	F	98
67	M	Secondary Acute Myeloid Leukemia	45	Unknown	M	100
25	M	Hodgkin's Lymphoma	42	27	F	100
65	F	Acute Myeloid Leukemia	62	44	M	99
61	M	Acute Myeloid Leukemia	13	Unknown	M	100
58	F	Secondary Acute Myeloid Leukemia	14	Unknown	M	100
48	F	Acute Lymphoblastic Leukemia	62	58	M	100

Table S4

SUPPLEMENTAL TABLE LEGENDS

Table S1. Multiplex PCR reactions and numbers of individuals (blood DNA samples) studied

WTCCC samples were screened with Plex 1 or Plex2. UKHLS samples were screened with Plex 3

* Only samples with ≥1000 reads at all studied loci were interrogated for the presence of mutations

Related to Table 1

Table S2. Read depth statistics for selected mutation calls in three recent studies using exome sequencing to identify individuals with ARCH

* Total read count (reference reads + mutant reads)

** SD = standard deviation

NB: These statistics are for coverage at called mutations. Numerical read depth (coverage) values for samples without identified mutations at these loci were not provided.

Related to Figure 1 & Table 2

Table S3. Characteristics of individuals sampled after autologous hematopoietic stem cell transplantation (HSCT)

Related to experimental procedures

Table S4. Characteristics of individuals sampled after allogeneic hematopoietic stem cell transplantation (HSCT) and their respective donors

Related to experimental procedures

Supplemental Experimental Procedures

Targeted re-sequencing

Multiplex primer combinations were tested and their concentrations adjusted to give similar levels of amplification for each of the target positions. First round multiplex PCR amplifications were performed with tailed gene primers on batches of up to 384 samples and individually barcoded by second round PCR with 384 pre-validated “MiSeq-ready” primers[1]; using a high fidelity polymerase (KAPA HiFi, Anachem or Q5 Hot Start HF, New England Biolabs). PCR reaction conditions used were as follows: 95°C for 3min, [98°C for 20s, 65°C for 60s, 60°C for 60s, 55°C for 60s, 50°C for 60s, 70°C for 60s] x6 cycles, held at 4°C until addition of barcoded second round primers, then [98°C for 20s, 62°C for 15s, 72°C for 30s] x19 cycles, 72°C 60s. For each batch, equal volumes of each sample were pooled, double SPRI size selected (X0.55 and X0.75) and quantified before storage at -20°C until sequencing. A total of 11 MiSeq runs (250nt paired-end) were used for mutation identification. One of the 11 sample batches was repeated from PCR to sequencing for experimental validation purposes (Supplemental Figure S2A). Also, the reproducibility of our assay in quantifying variant allele fractions (VAFs) was further confirmed by studying 11 unselected samples harboring *DNMT3A*-R882 mutant clones of varying sizes (VAF 0.01-0.18), using a different *DNMT3A*-R882 primer set in a singleplex PCR using the following conditions: 98° for 30s [98° for 20s, 60oC for 30s, 72°C for 60s] x6 cycles, held at 4oC until addition of barcoded second round primers, then [98°C for 20s, 62°C for 15s, 72°C for 30s] x19 cycles, 72°C 60s (Supplemental Figure S2B). Finally, the linearity of VAF calling was confirmed, using serial dilutions of leukemia or cell line DNA into cord blood DNA, for specific mutations including *DNMT3A*-R882, *JAK2*-V617F, *IDH1*-R132H, *IDH2*-R140Q, *SF3B1*-K666N, *SF3B1*-K700 and *NPM1*_mutation_A (TCTG duplication) (Supplemental figures S2C). The first 1571 samples (1531 WTCC, 32 post-transplant and 18 cord blood) were amplified using “Plex 1”, the next 1554 samples (1536 WTCC, and 18 cord blood) using “Plex 2” and the final 1152 samples (UKHLS) using Plex 3 primer sets.

Samples with two mutations at the same or neighboring loci

Amongst all samples, we identified 5 individuals with two independent spliceosome gene mutations of different VAFs (Figure 1B), 3 of which harbored the mutations at the same or at neighboring loci. Two of these, #760 and #565, harbored *SRSF2* P95H and *SRSF2* P95L, and another, #424, harbored *SF3B1* K666 and *SF3B1* K700. In an attempt to determine whether mutations were acquired on the same or on different alleles (maternal vs paternal), we looked for neighboring SNPs that could be used to “phase” the variants. We searched the Ensembl database for SNPs near *SRSF2* P95 and *SF3B1* K666/K700 and identified nearby polymorphisms for both locations, namely rs237057 (A/G MAF(G)=0.19) near *SRSF2* P95 and rs113023355 (A/G MAF(G)=0.012) near *SF3B1* K666 and K700. However, all three individuals were homozygous for the common alleles (A/A) and regrettably we were unable to phase the somatic variants.

Bioinformatic Analysis – Perl script for detecting *NPM1* mutations

In order to detect *NPM1* mutations with high sensitivity, we wrote a new Perl script to extract from each sample the reads covering the *NPM1* mutation hotspot and align these against the reference genome. Subsequently, the script individually parses each read looking for insertions at the hotspot position. The number of reads reporting the reference is recorded and so is the number reporting any variants and the sequence of this variant. Using sequencing data from normal samples manually spiked with *NPM1*-mutant DNA (OCI-AML3 cell line, mutation A), we determined that mutant reads with an expected VAF ≥ 0.002 (0.2%) were reliably detected (Supplemental Figure S2C).

Statistical Analysis and mutation calling threshold

We observed an apparent sequencing + PCR error rate < 0.13% after quality filters, which is broadly in line with sequencing errors observed elsewhere with current Illumina sequencing pipelines[2] and corresponds to a phred-scaled base call quality of 30. Postulating a binomial distribution of variant allele counts with this error probability and a total allele count (read depth) ≥ 1000, one would expect a false positive call rate $<10^{-5}$ when calling variants at VAF ≥ 0.008. To test this, we analyzed the range of VAFs derived from the study of 384 WTCC samples at 13 nucleotide positions at and around *DNMT3A* codon R882. Only amplicons giving ≥1000 reads were included in analyses. We found that the 3710 VAF values (10 positions x 371 samples) at positions outside the R882 hotspot (i.e. non-targets of known leukemia-associated mutations) were always ≤0.0045 indicating a very small combined PCR-MiSeq error rate. The 3 real subclonal samples in this group of 384 were easily distinguishable from error (Supplemental Figure S2D)

Supplemental References

1 Quail, M. A. *et al.* SASI-Seq: sample assurance Spike-Ins, and highly differentiating 384 barcoding for Illumina sequencing. *BMC genomics* **15**, 110, doi:10.1186/1471-2164-15-110 (2014).

2 Ekblom, R., Smeds, L. & Ellegren, H. Patterns of sequencing coverage bias revealed by ultra-deep sequencing of vertebrate mitochondria. *BMC genomics* **15**, 467, doi:10.1186/1471-2164-15-467 (2014).

Primer Name	Chromosome	Start coordinate (GRCh37)	Primer Sequence†
5247756-DNMT3A_p.R882_F	2	25457060	ACA CTC TTT CCC TAC ACG ACG CTC TTC CGA TCT CCT CAT GTT CTT GGT GTT TTmA T ‡
5247757-DNMT3A_p.R882_R	2	25457302	TCG GCA TTC CTG CTG AAC CGC TCT TCC GAT CTT TTT CTC CCC CAG GGT MTT mUG
5247759-IDH1_p.R132_F	2	209112927	ACA CTC TTT CCC TAC ACG ACG CTC TTC CGA TCT TAA ATG TGT GTA AAT ATA CAG TTmA T
5247760-IDH1_p.R132_R	2	209113173	TCG GCA TTC CTG CTG AAC CGC TCT TCC GAT CTR TTA TCT GCA AAA ATA TCY CmCC
5247762-IDH2_p.R140_R172_F	15	90631745	ACA CTC TTT CCC TAC ACG ACG CTC TTC CGA TCT AAG ARG ATG KCT AGG YGA GmGA
5247764-IDH2_p.R140_R172_R	15	90631986	TCG GCA TTC CTG CTG AAC CGC TCT TCC GAT CTC TCA MAG AGT TCA AGC TGA mAG
5247766-SRSF2_p.P95_F	17	74732797	ACA CTC TTT CCC TAC ACG ACG CTC TTC CGA TCT TGC TTC GCC GCG GAC CTT TmGT
5247767-SRSF2_p.P95_R	17	74733038	TCG GCA TTC CTG CTG AAC CGC TCT TCC GAT CTG AGG ACG CTA TGG ATG CCA mUG
5247768-SF3B1_p.K700_F	2	198266642	ACA CTC TTT CCC TAC ACG ACG CTC TTC CGA TCT TAG TAA TTT AGA TTT ATG TCG mCC
5247769-SF3B1_p.K700_R	2	198266886	TCG GCA TTC CTG CTG AAC CGC TCT TCC GAT CTG GCA TAG TTA AAA CCT GTG TmUT
5247770-SF3B1_p.K666_F	2	198267228	ACA CTC TTT CCC TAC ACG ACG CTC TTC CGA TCT ACC CTG TCT CCT AAA GAA AAmA A
5247771-SF3B1_p.K666_R	2	198267470	TCG GCA TTC CTG CTG AAC CGC TCT TCC GAT CTT AGA GCT TTT GCT GTT GTA mGC
5247772-NPM1_p.L287fsX_F	5	170837352	ACA CTC TTT CCC TAC ACG ACG CTC TTC CGA TCT TGT TTG GAA TTA AAT TAC ATC TmGA
5247773-NPM1_p.L287fsX_R	5	170837602	TCG GCA TTC CTG CTG AAC CGC TCT TCC GAT CTA AAA TTT TTT AAC AAA TTG TTT AAA mCT
5247774-repeat_CAG_F1	X	67545198	ACA CTC TTT CCC TAC ACG ACG CTC TTC CGA TCT AGG TGG ACC AGA AAT GGA AmAT
5247775-repeat_CAG_R1	X	67545441	TCG GCA TTC CTG CTG AAC CGC TCT TCC GAT CTT GTG GTC TTT ATC CAA AAG TTmU A
5739576-JAK2V617_F	9	5073696	ACA CTC TTT CCC TAC ACG ACG CTC TTC CGA TCT AGT CTT TCT TTG AAG CAG CAmA G
5739764-JAK2V617_R	9	5073887	TCG GCA TTC CTG CTG AAC CGC TCT TCC GAT CTA GTT TAC ACT GAC ACC TAG CmUG

Nucleotide sequences for multiplexed primers used in Plex 1

* Consecutive primers constitute forward (F) and reverse (R) primer pairs for the indicated loci

† Forward primers format: 5’ ACACTCTTTCCCTACACGACGCTCTTCCGATCT-[gene-specific forward] 3’, Reverse primerformat:5’ TCGGCATTCCTGCTGAACCGCTCTTCCGATCT-[gene-specific reverse] 3’

‡ "m" denotes a single 2'-O-Methyl base in place of the DNA base, used in order to minimise potential primer dimers

Primer Name*	Chromosome	Start coordinate (GRCh37)	Primer Sequence†
6029105-JAK2_V617_F	9	5073696	ACA CTC TTT CCC TAC ACG ACG CTC TTC CGA TCT AGT CTT TCT TTG AAG CAG CAmA G ‡
6029106-JAK2_V617_R	9	5073887	TCG GCA TTC CTG CTG AAC CGC TCT TCC GAT CTA GTT TAC ACT GAC ACC TAG CmUG
6029123-DNMT3A_R882_F	2	25457051	ACA CTC TTT CCC TAC ACG ACG CTC TTC CGA TCT TCT CTC CAT CCT CAT GTT CTmU G
6029124-DNMT3A_R882_R	2	25457284	TCG GCA TTC CTG CTG AAC CGC TCT TCC GAT CTT TGG TTT CCC AGT CCA CTA TmAC
6029109-TET2_H880_F	4	106157575	ACA CTC TTT CCC TAC ACG ACG CTC TTC CGA TCT AGT GCA TGC AAA ATA CAG GTmU T
6029110-TET2_H880_R	4	106157784	TCG GCA TTC CTG CTG AAC CGC TCT TCC GAT CTA ACT GAA GCT TGT TGT RAC TmUC
6029111-TET2_R1214_F	4	106164665	ACA CTC TTT CCC TAC ACG ACG CTC TTC CGA TCT TGA CCC TTG TTT TGT TTT GGmU T
6029112-TET2_R1214_F	4	106164877	TCG GCA TTC CTG CTG AAC CGC TCT TCC GAT CTT AAG CTC CGA GTA GAG TTT GmUC
6029113-KIT_exon8_F	4	55589690	ACA CTC TTT CCC TAC ACG ACG CTC TTC CGA TCT AGT GAA TGT TGC TGA GGT TTmU C
6029114-KIT_exon8_R	4	55589911	TCG GCA TTC CTG CTG AAC CGC TCT TCC GAT CTG TCC TTC CCC TCT GCA TTA TmAA
6029103-KIT_exon17_F	4	55599207	ACA CTC TTT CCC TAC ACG ACG CTC TTC CGA TCT TGG TTT TCT TTT CTC CTC CAmA C
6029104-KIT_exon17_R	4	55599396	TCG GCA TTC CTG CTG AAC CGC TCT TCC GAT CTT CCT TTG CAG GAC WGT CAmA G
6029115-NRAS_G12_F	1	115258606	ACA CTC TTT CCC TAC ACG ACG CTC TTC CGA TCT ATG GGT AAA GAT GAT CCG ACmA A
6029116-NRAS_G12_R	1	115258831	TCG GCA TTC CTG CTG AAC CGC TCT TCC GAT CTC GCC AAT TAA CCC TGA TTA CmUG
6029121-NRAS_Q61_F	1	115256340	ACA CTC TTT CCC TAC ACG ACG CTC TTC CGA TCT CCC TAG TGT GGT AAC CTC ATmU T
6029122-NRAS_Q61_R	1	115256573	TCG GCA TTC CTG CTG AAC CGC TCT TCC GAT CTA GAT GGT GAA ACC TGT TTG TmUR
6029107-KRAS_G12_F	12	25398214	ACA CTC TTT CCC TAC ACG ACG CTC TTC CGA TCT TGT TSG ATC ATA TTC RTC CAmC A
6029108-KRAS_G12_R	12	25398416	TCG GCA TTC CTG CTG AAC CGC TCT TCC GAT CTA AGG TAC TGG TGG AGT ATT TmGA
6029117-TET2_exon8_F	4	106182816	ACA CTC TTT CCC TAC ACG ACG CTC TTC CGA TCT TGG GAT TCA AAA TGT AAG GGmG A
6029118-TET2_exon8_R	4	106183041	TCG GCA TTC CTG CTG AAC CGC TCT TCC GAT CTT TGT TAC AAT TGC TGC CAA TmGA
6029119-FLT3_N676_F	13	28602158	ACA CTC TTT CCC TAC ACG ACG CTC TTC CGA TCT TGC TCA GTG TCT AAT TCC ACmU T
6029120-FLT3_N676_R	13	28602388	TCG GCA TTC CTG CTG AAC CGC TCT TCC GAT CTA GAA CTC AAG ATG ATG ACC CmAG
6029125-FLT3_D835_F	13	28592585	ACA CTC TTT CCC TAC ACG ACG CTC TTC CGA TCT TAG GAA ATA GCA GCC TCA CAmU T
6029126-FLT3_D835_F	13	28592819	TCG GCA TTC CTG CTG AAC CGC TCT TCC GAT CTG GTA CCT CCT ACT GAA GTT GmAG
6029127-ASXL1_F	20	31022393	ACA CTC TTT CCC TAC ACG ACG CTC TTC CGA TCT GGC GAG AGG TCA CCA CmUG
6029128-ASXL1_R	20	31022630	TCG GCA TTC CTG CTG AAC CGC TCT TCC GAT CTC TCC CYA TTT AGA GGA TAA GmGC
6029129-RUNX1_F	21	36252791	ACA CTC TTT CCC TAC ACG ACG CTC TTC CGA TCT TTT TGA AAT GTG GGT TTG TTmG C
6029130-RUNX1_R	21	36253035	TCG GCA TTC CTG CTG AAC CGC TCT TCC GAT CTC ATT TGT CCT TTG ACT GGT GmUT
NPM1_p.L287fsX_F	5	170837352	ACA CTC TTT CCC TAC ACG ACG CTC TTC CGA TCT TGT TTG GAA TTA AAT TAC ATC TmGA
NPM1_p.L287fsX_R	5	170837602	TCG GCA TTC CTG CTG AAC CGC TCT TCC GAT CTA AAA TTT TTT AAC AAA TTG TTT AAA mCT

Nucleotide sequences for multiplexed primers used in Plex 2

* Consecutive primers constitute forward (F) and reverse (R) primer pairs for the indicated loci

† Forward primers format: 5’ ACACTCTTTCCCTACACGACGCTCTTCCGATCT-[gene-specific forward] 3’, Reverse primerformat:5’ TCGGCATTCCTGCTGAACCGCTCTTCCGATCT-[gene-specific reverse] 3’

‡ "m" denotes a single 2'-O-Methyl base in place of the DNA base, used in order to minimise potential primer dimers

Primer Name*	Chromosome	Start coordinate (GRCh37)	Primer Sequence†
5247756-DNMT3A_p.R882_F	2	25457060	ACA CTC TTT CCC TAC ACG ACG CTC TTC CGA TCT CCT CAT GTT CTT GGT GTT TTmA T ‡
5247757-DNMT3A_p.R882_R	2	25457302	TCG GCA TTC CTG CTG AAC CGC TCT TCC GAT CTT TTT CTC CCC CAG GGT MTT mUG
5247759-IDH1_p.R132H_1_F	2	209112927	ACA CTC TTT CCC TAC ACG ACG CTC TTC CGA TCT TAA ATG TGT GTA AAT ATA CAG TTmA T
5247760-IDH1_p.R132H_1_R	2	209113173	TCG GCA TTC CTG CTG AAC CGC TCT TCC GAT CTR TTA TCT GCA AAA ATA TCY CmCC
5247762-IDH2_p.R140_R172_F	15	90631745	ACA CTC TTT CCC TAC ACG ACG CTC TTC CGA TCT AAG ARG ATG KCT AGG YGA GmGA
5247764-IDH2_p.R140_R172_R	15	90631986	TCG GCA TTC CTG CTG AAC CGC TCT TCC GAT CTC TCA MAG AGT TCA AGC TGA mAG
5247766-SRSF2_p.P95_F	17	74732797	ACA CTC TTT CCC TAC ACG ACG CTC TTC CGA TCT TGC TTC GCC GCG GAC CTT TmGT
5247767-SRSF2_p.P95_R	17	74733038	TCG GCA TTC CTG CTG AAC CGC TCT TCC GAT CTG AGG ACG CTA TGG ATG CCA mUG
5247768-SF3B1_p.K700_F	2	198266642	ACA CTC TTT CCC TAC ACG ACG CTC TTC CGA TCT TAG TAA TTT AGA TTT ATG TCG mCC
5247769-SF3B1_p.K700_R	2	198266886	TCG GCA TTC CTG CTG AAC CGC TCT TCC GAT CTG GCA TAG TTA AAA CCT GTG TmUT
5247770-SF3B1_p.K666_F	2	198267228	ACA CTC TTT CCC TAC ACG ACG CTC TTC CGA TCT ACC CTG TCT CCT AAA GAA AAmA A
5247771-SF3B1_p.K666_R	2	198267470	TCG GCA TTC CTG CTG AAC CGC TCT TCC GAT CTT AGA GCT TTT GCT GTT GTA mGC
5247772-NPM1_p.L287fsX_F	5	170837352	ACA CTC TTT CCC TAC ACG ACG CTC TTC CGA TCT TGT TTG GAA TTA AAT TAC ATC TmGA
5247773-NPM1_p.L287fsX_R	5	170837602	TCG GCA TTC CTG CTG AAC CGC TCT TCC GAT CTA AAA TTT TTT AAC AAA TTG TTT AAA mCT
5739576-JAK2V617_F	9	5073696	ACA CTC TTT CCC TAC ACG ACG CTC TTC CGA TCT AGT CTT TCT TTG AAG CAG CAmA G
5739764-JAK2V617_R	9	5073887	TCG GCA TTC CTG CTG AAC CGC TCT TCC GAT CTA GTT TAC ACT GAC ACC TAG CmUG
6029103-KIT_exon17_F	4	55599207	ACA CTC TTT CCC TAC ACG ACG CTC TTC CGA TCT TGG TTT TCT TTT CTC CTC CAmA C
6029104-KIT_exon17_R	4	55599396	TCG GCA TTC CTG CTG AAC CGC TCT TCC GAT CTT CCT TTG CAG GAC WGT CAmA G
6029107-KRAS_G12_F	12	25398214	ACA CTC TTT CCC TAC ACG ACG CTC TTC CGA TCT TGT TSG ATC ATA TTC RTC CAmC A
6029108-KRAS_G12_R	12	25398416	TCG GCA TTC CTG CTG AAC CGC TCT TCC GAT CTA AGG TAC TGG TGG AGT ATT TmGA
6029115-NRAS_G12_F	1	115258606	ACA CTC TTT CCC TAC ACG ACG CTC TTC CGA TCT ATG GGT AAA GAT GAT CCG ACmA A
6029116-NRAS_G12_R	1	115258831	TCG GCA TTC CTG CTG AAC CGC TCT TCC GAT CTC GCC AAT TAA CCC TGA TTA CmUG
6029121-NRAS_Q61_F	1	115256340	ACA CTC TTT CCC TAC ACG ACG CTC TTC CGA TCT CCC TAG TGT GGT AAC CTC ATmU T
6029122-NRAS_Q61_R	1	115256573	TCG GCA TTC CTG CTG AAC CGC TCT TCC GAT CTA GAT GGT GAA ACC TGT TTG TmUR
6029125-FLT3_D835_F	13	28592585	ACA CTC TTT CCC TAC ACG ACG CTC TTC CGA TCT TAG GAA ATA GCA GCC TCA CAmU T
6029126-FLT3_D835_R	13	28592819	TCG GCA TTC CTG CTG AAC CGC TCT TCC GAT CTG GTA CCT CCT ACT GAA GTT GmAG

Nucleotide sequences for multiplexed primers used in Plex 3

* Consecutive primers constitute forward (F) and reverse (R) primer pairs for the indicated loci

† Forward primers format: 5’ ACACTCTTTCCCTACACGACGCTCTTCCGATCT-[gene-specific forward] 3’, Reverse primerformat:5’ TCGGCATTCCTGCTGAACCGCTCTTCCGATCT-[gene-specific reverse] 3’

‡ "m" denotes a single 2'-O-Methyl base in place of the DNA base, used in order to minimise potential primer dimers
