# Supplementary material for: Leukemia-Associated Somatic Mutations Drive Distinct Patterns of Age-Related Clonal Hemopoiesis
Source: Cell Rep. 2015 Feb 26;10(8):1239–45. doi: 10.1016/j.celrep.2015.02.005 (PMC4542313; doi:10.1016/j.celrep.2015.02.005)
Supplement: Document S2. Article plus Supplemental Information [file mmc2.pdf]

Cell Reports

Report

Leukemia-Associated Somatic Mutations Drive Distinct Patterns of Age-Related Clonal Hemopoiesis

Graphical Abstract

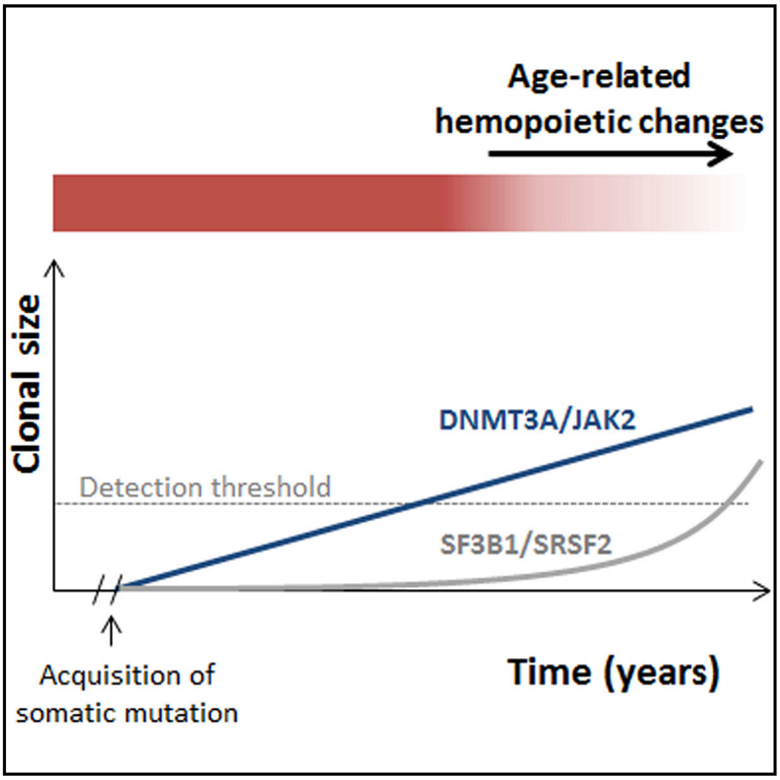

Highlights

- Clonal hemopoiesis is an almost inevitable consequence of aging in humans
- Spliceosome gene mutations drove clonal hemopoiesis only in persons aged ≥70 years
- NPM1 mutations behave as gatekeepers for leukemogenesis

Authors

Thomas McKerrell, Naomi Park, ..., Ignacio Varela, George S. Vassiliou

Correspondence

gsv20@sanger.ac.uk

In Brief

McKerrell et al. employ ultra-deep sequencing to show that age-related clonal hemopoiesis is much more common than previously realized. They find that clonal hemopoiesis, driven by mutations in spliceosome genes *SF3B1* and *SRSF2*, was noted exclusively in individuals aged 70 years or older and that *NPM1* mutations are not seen in association with this phenomenon, endorsing their close association with leukemogenesis.

McKerrell et al., 2015, Cell Reports *10*, 1239–1245
March 3, 2015 ©2015 The Authors
http://dx.doi.org/10.1016/j.celrep.2015.02.005

Cell Reports
Report

OPEN ACCESS
CellPress

Leukemia-Associated Somatic Mutations Drive Distinct Patterns of Age-Related Clonal Hemopoiesis

Thomas McKerrell,[1,13] Naomi Park,[2,13] Thaidy Moreno,[3] Carolyn S. Grove,[1] Hannes Ponstingl,[1] Jonathan Stephens,[4,5] Understanding Society Scientific Group,[6] Charles Crawley,[7] Jenny Craig,[7] Mike A. Scott,[7] Clare Hodkinson,[4,8] Joanna Baxter,[4,8] Roland Rad,[9,10] Duncan R. Forsyth,[11] Michael A. Quail,[2] Eleftheria Zeggini,[12] Willem Ouwehand,[4,5,12] Ignacio Varela,[3] and George S. Vassiliou[1,4,7,*]
[1]Haematological Cancer Genetics, Wellcome Trust Sanger Institute, Cambridge CB10 1SA, UK
[2]Sequencing Research Group, Wellcome Trust Sanger Institute, Cambridge CB10 1SA, UK
[3]Instituto de Biomedicina y Biotecnología de Cantabria (CSIC-UC-Sodercan), Departamento de Biología Molecular, Universidad de Cantabria, 39011 Santander, Spain
[4]Department of Haematology, Cambridge Biomedical Campus, University of Cambridge, Cambridge CB2 0XY, UK
[5]NHS Blood and Transplant, Cambridge Biomedical Campus, Cambridge CB2 0PT, UK
[6]Institute for Social and Economic Research, University of Essex, Colchester CO4 3SQ, UK
[7]Department of Haematology, Cambridge University Hospitals NHS Trust, Cambridge CB2 0QQ, UK
[8]Cambridge Blood and Stem Cell Biobank, Department of Haematology, University of Cambridge, Cambridge CB2 0XY, UK
[9]Department of Medicine II, Klinikum Rechts der Isar, Technische Universität München, 81675 München, Germany
[10]German Cancer Consortium (DKTK), German Cancer Research Center (DKFZ), 69120 Heidelberg, Germany
[11]Department of Medicine for the Elderly, Cambridge University Hospitals NHS Trust, Cambridge CB2 0QQ, UK
[12]Human Genetics, Wellcome Trust Sanger Institute, Cambridge CB10 1SA, UK
[13]Co-first author
*Correspondence: gsv20@sanger.ac.uk
http://dx.doi.org/10.1016/j.celrep.2015.02.005
This is an open access article under the CC BY license (http://creativecommons.org/licenses/by/3.0/).

SUMMARY

Clonal hemopoiesis driven by leukemia-associated gene mutations can occur without evidence of a blood disorder. To investigate this phenomenon, we interrogated 15 mutation hot spots in blood DNA from 4,219 individuals using ultra-deep sequencing. Using only the hot spots studied, we identified clonal hemopoiesis in 0.8% of individuals under 60, rising to 19.5% of those ≥90 years, thus predicting that clonal hemopoiesis is much more prevalent than previously realized. *DNMT3A*-R882 mutations were most common and, although their prevalence increased with age, were found in individuals as young as 25 years. By contrast, mutations affecting spliceosome genes *SF3B1* and *SRSF2*, closely associated with the myelodysplastic syndromes, were identified only in those aged >70 years, with several individuals harboring more than one such mutation. This indicates that spliceosome gene mutations drive clonal expansion under selection pressures particular to the aging hemopoietic system and explains the high incidence of clonal disorders associated with these mutations in advanced old age.

INTRODUCTION

Cancers develop through the combined action of multiple mutations that are acquired over time (Nowell, 1976). This paradigm is well established in hematological malignancies, whose clonal history can be traced back for several years or even decades (Ford et al., 1998; Kyle et al., 2002). It is also clear from studies of paired diagnostic-relapsed leukemia samples that recurrent disease can harbor some, but not always all, mutations present at diagnosis, providing evidence for the presence of a clone of ancestral pre-leukemic stem cells that escape therapy and give rise to relapse through the acquisition of new mutations (Ding et al., 2012; Krönke et al., 2013). Studies of such phenomena have defined a hierarchical structure among particular leukemia mutations, with some, such as those affecting the gene *DNMT3A*, displaying the characteristics of leukemia-initiating lesions and driving the expansion of hemopoietic cell clones prior to the onset of leukemia (Ding et al., 2012; Shlush et al., 2014).

These observations suggest that individuals without overt features of a hematological disorder may harbor hemopoietic cell clones carrying leukemia-associated mutations. In fact, such mutations, ranging from large chromosomal changes (Jacobs et al., 2012; Laurie et al., 2012) to nucleotide substitutions (Busque et al., 2012), have been found to drive clonal hemopoiesis in some individuals. Recent reanalyses of large exome-sequencing data sets of blood DNA showed that clonal hemopoiesis is more common than previously realized and increases with age to affect up to 11% of those over 80 and 18.4% of those over 90 years (Genovese et al., 2014; Jaiswal et al., 2014; Xie et al., 2014). The presence of such clones was associated with an increased risk of developing hematological or other cancers and a higher all-cause mortality, probably due to an increased risk of cardiovascular disease (Genovese et al., 2014; Jaiswal et al., 2014).

CrossMark

Table 1. Mutation Hot Spots Interrogated in This Study

Gene	Target Codon
DNMT3A	R882
JAK2	V617
NPM1	L287
SRSF2	P95
SF3B1	K666
SF3B1	K700
IDH1	R132
IDH2	R140
IDH2	R172
KRAS	G12
NRAS	G12
NRAS	Q61
KIT	D816
FLT3	D835
FLT3	N676

Also see Table S1 for detailed information about numbers of samples screened for each mutation.

The important findings of these studies were based on analysis of exome-sequencing data sets that were generated for the study of constitutional genomes, thus trading genome-wide coverage for reduced sensitivity for detecting small subclonal events. We used the different approach of targeted re-sequencing of selected leukemia-associated mutation hot spots in blood DNA from more than 4,000 individuals unselected for blood disorders. In addition to increasing the sensitivity for detecting subclonal mutations, this approach enabled us to prospectively select and study a large number of elderly individuals. Our results show that clonal hemopoiesis is significantly more common than anticipated, give new insights into the distinct age-distribution and biological behavior of clonal hemopoiesis driven by different mutations, and help explain the increased incidence of myelodysplastic syndromes (MDSs) with advancing age.

RESULTS

To investigate the incidence, target genes, and age distribution of age-related clonal hemopoiesis (ARCH), we performed targeted re-sequencing for hot spot mutations at 15 gene loci recurrently mutated in myeloid malignancies (Table 1) using blood DNA from 3,067 blood donors aged 17–70 (Wellcome Trust Case Control Consortium [WTCCC]) and 1,152 unselected individuals aged 60–98 years (United Kingdom Household Longitudinal Study [UKHLS]; see Figure S1 for detailed age distributions). To do this, we developed and validated a robust methodology, employing barcoded multiplex PCR of mutational hot spots followed by next-generation sequencing (MiSeq) and bioinformatic analysis, to extract read counts and allelic fractions for reference and non-reference nucleotides. This reliably detected mutation-associated circulating blood cell clones with a variant allele fraction (VAF) $\geq$ 0.008 (0.8%; see Supplemental Experimental Procedures and Figure S2).

We obtained adequate coverage ($\geq$1,000 reads at all studied hot spots) from 4,067 blood DNA samples and identified mutation-bearing clones in 105 of these. Of note, not all hot spots were studied in all samples and the derived incidence of mutations in our population as a whole was 3.24% (Table S1). However, the incidence rose significantly with age from 0.2% in the 17–29 to 19.5% in the 90–98 years age group (Figure 1A). We found one or more samples with mutations at 9 of the 15 hot spot codons studied, with VAFs varying widely within and between mutation groups (Table 2).

The most-common mutations were those affecting *DNMT3A* R882, whose incidence rose with age from 0.2% (1/489) in the 17–25 to a peak of 3.1% (11/355) in the 80–89 age group. A similar pattern was observed with *JAK2* V617F mutations (Figure 1A). By contrast, spliceosome gene mutations at *SRSF2* P95, *SF3B1* K666, and *SF3B1* K700 were exclusively observed in people aged over 70 years, rising sharply from 1.8% in those aged 70–79 to 8.3% in the 90–98 years age group. Among all samples, we identified only six individuals with more than one mutation; significantly, five of them had two independent spliceosome gene mutations of different VAFs (Figure 1B). Unfortunately, in each of three cases with two mutations at the same or nearby positions, neighboring SNPs were not informative and the variants could not be phased (see Supplemental Experimental Procedures).Occasional mutations in the genes *IDH1*, *IDH2*, *NRAS*, and *KRAS* were also seen. Except for three samples with IDH1/2 mutations, hemoglobin concentrations did not differ significantly between individuals with and without hot spot mutations (Figure S3A). For samples with full blood count results available, *JAK2* V617F mutant cases had a higher platelet count (albeit within the normal range) than "no mutation cases," whereas other results did not differ (Figure S3B). No hot spot mutations were found in the few cord blood (n = 18) and post-transplantation (n = 32) samples studied.

Finally, despite using a very sensitive method and a mutation-calling script written specifically for this purpose, no samples with *NPM1* mutations of VAF $\geq$ 0.008 were identified. In fact, variant reads reporting a canonical *NPM1* mutation (mutation A; TCTG duplication) were detected in only 1 of 4,067 samples at a VAF of 0.0012 (4/3,466 reads).

DISCUSSION

Hematological malignancies develop through the serial acquisition of somatic mutations in a process that can take many years or even decades (Ford et al., 1998; Kyle et al., 2002). Also, it is clear that the presence of hemopoietic cells carrying leukemia-associated mutations is only followed by the onset of hematological malignancies in a minority of cases (Busque et al., 2012; Genovese et al., 2014; Jacobs et al., 2012; Jaiswal et al., 2014; Laurie et al., 2012; Xie et al., 2014). In order to understand the incidence and clonal dynamics of pre-leukemic clonal hemopoiesis, we interrogated 15 leukemia-associated mutation hot spots using a highly sensitive methodology able to detect small clones with mutations.

We show that clonal hemopoiesis is rare in the young but becomes common with advancing age. In particular, we observed that ARCH driven by the mutations studied here doubled in

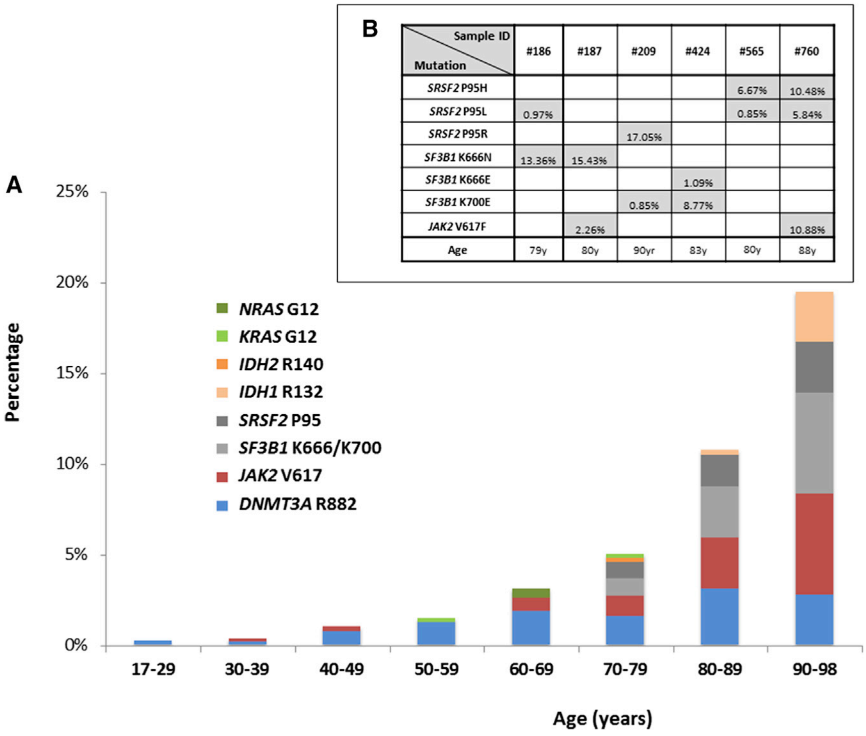

Sample ID / Mutation	#186	#187	#209	#424	#565	#760
SRSF2 P95H					6.67%	10.48%
SRSF2 P95L	0.97%				0.85%	5.84%
SRSF2 P95R			17.05%			
SF3B1 K666N	13.36%	15.43%				
SF3B1 K666E				1.09%		
SF3B1 K700E			0.85%	8.77%		
JAK2 V617F		2.26%				10.88%
Age	79y	80y	90yr	83y	80y	88y

Figure 1. Prevalence and Age Distribution of Hot Spot Mutations Driving Clonal Hemopoiesis

(A) Prevalence of mutations driving clonal hemopoiesis by age.

(B) Samples with more than one mutation, variant allele fraction (VAF) of each mutation present, and age of participant.

Also see Figure S1 for age distribution of all participants.

frequency in successive decades after the age of 50, rising from 1.5% in those aged 50–59 to 19.5% in those aged 90–98 (Figure 1). Of note, 61 of 112 clones identified had a VAF $\leq$ 3% (Table 2), and it is likely that most of these would not have been detected by conventional exome sequencing, which gives lower than 10-fold average coverage compared to the current study (see Table S2 for comparison to such studies), with some recurrently mutated regions giving particularly low coverage (Genovese et al., 2014). Notably, our study did not search for non-hot-spot mutations associated with ARCH such as those affecting genes *TET2* and *ASXL1* or *DNMT3A* codons other than R882 (Genovese et al., 2014; Jaiswal et al., 2014; Xie et al., 2014). Assuming that the incidence of small clones is similar for such mutations as for the hot spot mutations we studied here, the mean projected true incidence of ARCH driven by leukemia-associated mutations in those older than 90 years is greater than 70% (Figure S4). This makes clonal hemopoiesis an almost inevitable consequence of advanced aging.

Another significant finding of our study is the disparate age distribution of ARCH associated with different mutation types. In particular, we found that, although *DNMT3A* R882 and *JAK2* V617F mutations become more common with age, they were also found in younger individuals. This is in keeping with the increasing cumulative likelihood of their stochastic acquisition with the passage of time. In contrast, spliceosome gene mutations were found exclusively in those aged 70 years or older, replicating the sharp rise beyond this age in the incidence of MDSs driven by these mutations and the fact that, among unselected MDS patients, those with spliceosome mutations are significantly older than those without (Haferlach et al., 2014; Lin et al., 2014; Papaemmanuil et al., 2013; Wu et al., 2012). Exome-sequencing studies describe a much-lower rate of spliceosome mutations (Genovese et al., 2014; Jaiswal et al., 2014; Xie et al., 2014), but this is again likely to reflect their lower sensitivity for detecting small clones, which was a particular limitation at spliceosome mutation hot spots as these were captured/sequenced at lower-than-average depths (Table S2). In our study, 19/33 *SF3B1*- or *SRSF2*-associated clones had a VAF $\leq$ 5%, with 13 of these at VAFs $\leq$ 3% (Table 2), the majority of which would not have been detected by low-coverage sequencing. The identification of ARCH driven by spliceosome gene mutations is in keeping with the fact that these are founding mutations in the clonal evolution of MDS and related hematological malignancies (Cazzola et al., 2013; Haferlach et al., 2014; Papaemmanuil et al., 2013).

We propose that the exclusive identification of spliceosome gene mutations in those aged $\geq$70 years can be explained by differences in the prevailing pressures on clonal selection at different ages, which can in turn explain how different gene mutations can generate detectable clonal expansions at different ages (Figure 2). The alternatives are that spliceosome mutations are associated with slower rates of clonal expansion or that they are detected later because they contribute less to circulating leukocytes. Both of these scenarios are less plausible, given the complete absence of such mutations even at low VAFs in younger age groups. For any somatic mutation imparting a clonal advantage to a stem/progenitor cell and leading to the generation of a steadily expanding clone, one would expect such a clone to be detectable at a smaller size at earlier and a larger size at later time points, as is the case for *DNMT3A* R882 and *JAK2* V617 mutations. Instead, clones (of any size) driven by mutant *SRSF2* and *SF3B1* were observed exclusively in individuals aged 70 years or older, suggesting that these only begin to expand later in life. Furthermore, considerable support for the presence of a different selection milieu comes from the observation that five of six patients with multiple mutations harbored two independent spliceosome gene mutations, indicative of convergent evolution, i.e., evolution to overcome a shared selective pressure or to exploit a shared environment (Greaves and Maley, 2012; Rossi et al., 2008).

It is tempting to consider the nature of age-related changes in normal hemopoiesis that make it permissive to the outgrowth of

Table 2. Amino Acid Consequences and VAFs of the 112 Clonal Mutations Identified in This Study

Mutation Hot Spot	Codon	VAF (%)	Age	Mutation Hot Spot	Codon	VAF (%)	Age	Mutation Hot Spot	Codon	VAF (%)	Age
DNMT3A R882	p.R882H	4.14	25		p.R882H	32.02	81	*IDH1 R132*	p.R132H	42.13	84
	p.R882C	2.33	35		p.R882H	1.14	81		p.R132C	0.92	92
	p.R882H	3.80	42		p.R882H	3.06	81	*IDH2 R140*	p.R140Q	6.67	76
	p.R882H	4.00	42		p.R882H	2.17	81	*SRSF2 P95*	p.P95R	4.46	70
	p.R882H	1.25	43		p.R882H	1.13	82		p.P95L	3.35	72
	p.R882H	19.00	48		p.R882H	1.46	82		p.P95H	0.86	73
	p.R882H	1.18	49		p.R882C	2.62	82		p.P95H	0.84	77
	p.R882S	1.74	49		p.R882C	6.15	89		p.P95L	0.97	79†
	p.R882H	9.87	50		p.R882C	2.00	94		p.P95L	0.85	80††
	p.R882H	0.83	51	*JAK2V617F*	p.V617F	1.56	34		p.P95H	6.67	80††
	p.R882C	1.10	51		p.V617F	4.91	42		p.P95L	0.96	81
	p.R882C	12.50	52		p.V617F	7.72	45		p.P95H	6.40	82
	p.R882C	1.28	53		p.V617F	0.85	62		p.P95L	2.74	85
	p.R882C	2.47	54		p.V617F	25.44	64		p.P95R	7.52	87
	p.R882H	1.95	55		p.V617F	7.41	65		p.P95L	5.84	88**
	p.R882C	30.22	55		p.V617F	1.03	67		p.P95H	10.48	88**
	p.R882C	1.22	56		p.V617F	0.88	71		p.P95R	2.71	88
	p.R882H	0.91	58		p.V617F	3.75	71		p.P95R	17.05	90‡
	p.R882H	4.17	60		p.V617F	1.16	75	*SF3B1* K700	p.K700E	1.04	76
	p.R882H	5.90	60		p.V617F	2.30	77		p.K700E	6.63	81
	p.R882H	9.60	60		p.V617F	1.92	78		p.K700E	0.79	82
	p.R882H	2.73	60		p.V617F	2.26	80*		p.K700E	12.59	83
	p.R882C	9.33	60		p.V617F	4.25	80		p.K700E	8.77	83‡‡
	p.R882H	7.03	61		p.V617F	1.92	80		p.K700E	1.02	84
	p.R882C	1.21	61		p.V617F	3.71	80		p.K700E	0.85	90‡
	p.R882H	0.86	63		p.V617F	15.48	81		p.K700E	1.37	90
	p.R882H	2.54	64		p.V617F	1.21	82	*SF3B1* K666	p.K666N	1.33	70
	p.R882H	3.19	67		p.V617F	1.62	85		p.K666N	5.01	79
	p.R882H	2.74	70		p.V617F	0.83	85		p.K666N	13.36	79†
	p.R882H	4.27	74		p.V617F	1.98	86		p.K666N	15.43	80*
	p.R882H	0.85	74		p.V617F	25.94	88		p.K666N	4.60	81
	p.R882H	0.85	75		p.V617F	10.88	88**		p.K666E	1.09	83‡‡
	p.R882C	1.12	77		p.V617F	2.94	90		p.K666N	35.11	86
	p.R882C	1.15	78		p.V617F	1.23	90		p.K666N	19.70	86
	p.R882H	1.26	79	*KRAS G12*	p.G12 R	0.94	55		p.K666N	16.55	86
	p.R882H	16.66	80		p.G12S	2.78	78		p.K666E	3.34	95
	p.R882C	4.28	80	*NRAS G12*	p.G12S	1.50	61				
	p.R882C	3.66	80		p.G12D	0.96	62				

Mutations identified in the same sample are highlighted with the same symbol (*, **, †, ††, ‡, and ‡‡).

clones driven by spliceosome mutations. HSCs do not operate in isolation; instead, their normal survival and behavior are closely dependent on interactions with the hemopoietic microenvironment (Calvi et al., 2003; Rossi et al., 2008; Zhang et al., 2003). Therefore, both cell-intrinsic and microenvironmental factors influence hemopoietic aging (Rossi et al., 2008; Woolthuis et al., 2011). For example, there is good evidence for age-related changes in cell-intrinsic properties of HSCs in both mice (Chambers et al., 2007; Rossi et al., 2005) and humans (Rübe et al., 2011; Taraldsrud et al., 2009), and it is also clear that aging has a profound effect on the hemopoietic niche, reducing its ability to sustain polyclonal hemopoiesis, favoring oligo- or monoclonality instead (Vas et al., 2012). These and many other observations provide strong evidence that changes in the hemopoietic system subject HSCs to changing pressures during normal aging, driving clonal selection (Rossi et al., 2008).

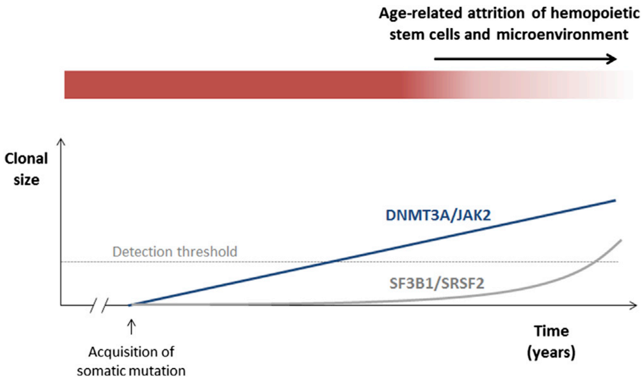

Figure 2. Proposed Kinetics of Hemopoietic Clones Driven by Different Gene Mutations

Mutations such as *DNMT3A* R882H/C or *JAK2* V617F drive a slow but inexorable clonal expansion, leading to the outgrowth of a detectable clone after a certain latency. By contrast, mutations affecting spliceosome genes, such as *SF3B1* and *SRSF2*, and acquired at the same age for the purposes of this model give no proliferative advantage initially but do so later in the context of an aging hemopoietic compartment. Their effects may operate by prolonging stem cell survival and repopulating fitness beyond that of normal stem cells or by exploiting cell-extrinsic changes in the aging microenvironment.

A striking example of such selection was described in a 115-year-old woman whose peripheral white blood cells were shown to be primarily the offspring of only two related HSC clones, whose cargo of approximately 450 somatic mutations did not include known leukemogenic mutations (Holstege et al., 2014). In the absence of somatic driver mutations, it is probable that such selection is driven by well-demonstrated epigenetic differences between individual HSCs (Fraga et al., 2005) or by stochastic events. Furthermore, clonal hemopoiesis in the absence of a known leukemia-driver mutation was also well documented recently (Genovese et al., 2014), and whereas unknown or undetected drivers may be responsible for many cases of this phenomenon, it is also highly plausible that a stochastic process of clonal selection or loss may operate in others. Our study provides evidence that spliceosome gene mutations offer a means to exploit age-related changes in hemopoiesis to drive clonal hemopoiesis in advanced old age, an observation that blurs the boundary between "driver" and "passenger" mutations. Such a context dependency is not a surprising attribute for the effects of spliceosome mutations, which have not, so far, been shown to impart a primary proliferative advantage to normal hemopoietic stem and progenitor cells (Matsunawa et al., 2014; Visconte et al., 2012).

A final important finding of our study was the almost complete absence of canonical *NPM1* mutations in our collection of more than 4,000 people, despite the use of a highly sensitive assay for their detection, designed specifically for this study. Among more than 10 million mapped reads covering this mutation hot spot, we identified only four reads in a single sample reporting a canonical mutation (mutation A; TCTG duplication). Given their frequency in myeloid leukemia (Cancer Genome Atlas Research Network, 2013) and the fact that they are not late mutations (Krönke et al., 2013; Shlush et al., 2014), this observation frames *NPM1* mutations as "gatekeepers" of leukemogenesis, i.e., their acquisition appears to be closely associated with the development of frank leukemia. In this light, the frequent co-occurrence of *DNMT3A* and *NPM1* mutations suggests that the former behave as "rafts" that enable *NPM1* mutant clones to be founded and expanded, thus facilitating onward evolution toward acute myeloid leukemia.

We used a highly sensitive method to search for evidence of clonal hemopoiesis driven by 15 recurrent leukemogenic mutations in more than 4,000 individuals. Our results demonstrate that the incidence of clonal hemopoiesis is much higher than suggested by exome-sequencing studies, that spliceosome gene mutations drive clonal outgrowth primarily in the context of an aging hemopoietic compartment, and that *NPM1* mutations do not drive ARCH, indicating that their acquisition is closely associated with frank leukemia.

EXPERIMENTAL PROCEDURES

Patient Samples

Samples were obtained with written informed consent and in accordance with the Declaration of Helsinki and appropriate ethics committee approvals from all participants (approval reference numbers 10/H0604/02, 07/MRE05/44, and 05/Q0106/74). Maternal consent was obtained for the use of cord blood samples. Samples were obtained from 3,067 blood donors aged 17–70 years (WTCCC; UK Blood Services 1 [UKBS1] and UKBS2 common controls), 1,152 unselected individuals aged 60–98 years (UKHLS; https://www.understandingsociety.ac.uk/), 32 patients that had undergone a hemopoietic stem cell transplant (12 autologous and 20 allogeneic; Tables S3 and S4) 1 month to 14 years previously, and 18 cord blood samples. Age distribution of the WTCCC and UKHLS cohorts/samples is shown in Figure S1. Hemoglobin concentrations were available for a total of 3,587 of the 4,067 samples from which adequate sequencing data were obtained for analysis, including 102 of 105 samples with mutations. Full blood count results were available for 2,952 WTCCC samples. The average blood donation frequency for WTCCC donors was 1.6 donations of one unit per year. Details of donations by individual participants were not available.

Targeted Sequencing

Genomic DNA was used to simultaneously amplify several gene loci using multiplex PCR, in order to capture and analyze 15 mutational hot spots enriched for, but not exclusive to, targets of mutations thought to arise early in leukemogenesis (Table 1). We used three multiplex primer combinations (Plex1-3), guided by our findings, to capture the targeted mutational hot spots (Table S1). Primers were designed using the Hi-Plex PCR-MPS (massively parallel sequencing) strategy (Nguyen-Dumont et al., 2013), except for *JAK2* V617 and "Plex2" primers, which were designed using MPRIMER (Shen et al., 2010). These and additional primer sequences used in each Plex and details of PCR- and DNA-sequencing protocols are detailed in Supplemental Experimental Procedures. Methodological validation experiments are shown in Figure S2.

Bioinformatic Analysis

Sequencing data were aligned to the human reference genome (hg19) using BWA. Subsequently, the SAMTOOLS pileup command was used to generate pileup files from the generated bam files (version 0.1.8; http://samtools.sourceforge.net; Li et al., 2009). A flexible in-house Perl script generated by our group, MIDAS (Conte et al., 2013), was modified in order to interrogate only the hot spot nucleotide positions of interest (those with reported mutations in the COSMIC database; Forbes et al., 2015) on the pileup file, considering only those reads with a sequence quality higher than 25 and a mapping quality higher than 15. For each sample, the numbers of reads reporting the reference and variant alleles at each position were extracted. VAFs were derived by dividing the number of reads reporting the most-frequent variant nucleotide to the total. In order to detect NPM1 mutations with high sensitivity,

we wrote a bespoke Perl script described in Supplemental Experimental Procedures.

Statistical Analyses and Mutation-Calling Threshold

We chose a threshold VAF of $\geq$0.008 (0.8%) to "call" clones with a heterozygous mutation representing $\geq$1.6% of blood leukocytes. From validation experiments and data analysis (see Supplemental Experimental Procedures and Figure S2D), we determined that the maximum false-positive error rate for calling a mutation (VAF $\geq$ 0.008) due to variant allele counts that are solely due to PCR-MiSeq error was negligible ($p < 10^{-5}$). For comparisons of blood cell counts and hemoglobin concentrations, we used non-paired t tests. For summary statistics of read coverage (Table S2) and for the purposes of deriving an estimate of the overall incidence of clonal hemopoiesis (Figure S4), we used published tables of all mutations reported by three recent studies that employed whole-exome-sequencing analyses to identify individuals with clonal hemopoiesis (Genovese et al., 2014; Jaiswal et al., 2014; Xie et al., 2014).

ACCESSION NUMBERS

The European Genome-Phenome Archive (EGA) accession number for the sequencing data reported in this paper is EGAS00001000814.

SUPPLEMENTAL INFORMATION

Supplemental Information includes Supplemental Experimental Procedures, four figures, and four tables and can be found with this article online at http://dx.doi.org/10.1016/j.celrep.2015.02.005.

AUTHOR CONTRIBUTIONS

G.S.V. conceived and designed the study. G.S.V. and T. McKerrell supervised the study, analyzed data, and wrote the manuscript. N.P. and T. McKerrell performed experimental procedures. I.V. and T. Moreno wrote scripts and performed bioinformatics analysis. H.P., T. McKerrell, and G.S.V. performed statistical analyses. E.Z., C.S.G., M.A.Q., and R.R. contributed to study strategy and to technical and analytical aspects. U.S.S.G., E.Z., W.O, J.C., C.C., J.B., J.S., C.H., M.A.S., and D.R.F. contributed to sample acquisition and subject recruitment.

ACKNOWLEDGMENTS

This project was funded by a Wellcome Trust Clinician Scientist Fellowship (100678/Z/12/Z; to T. McKerrell) and by the Wellcome Trust Sanger Institute (grant number WT098051). G.S.V. is funded by a Wellcome Trust Senior Fellowship in Clinical Science (WT095663MA), and work in his laboratory is also funded by Leukaemia Lymphoma Research and the Kay Kendal Leukaemia Fund. I.V. is funded by Spanish Ministerio de Economía y Competitividad subprograma Ramón y Cajal. C.S.G. is funded by a Leukaemia Lymphoma Research Clinical Research Training Fellowship. We thank Servicio Santander Supercomputación for their support. We acknowledge use of DNA from The UK Blood Services Collection of Common Controls (UKBS collection), funded by the Wellcome Trust grant 076113/C/04/Z, by the Juvenile Diabetes Research Foundation grant WT061858, and by the National Institute of Health Research of England. The collection was established as part of the Wellcome Trust Case-Control Consortium. We also gratefully acknowledge use of blood DNA samples and data from participants of the UK Household Longitudinal Study (https://www.understandingsociety.ac.uk/), collected by NatCen and the Institute for Social and Economic Research, University of Essex, and funded by the Economic and Social Research Council, UK. We thank the Cambridge Blood and Stem Cell Biobank and the Cancer Molecular Diagnosis Laboratory, Cambridge Biomedical Research Centre (National Institute for Health Research, UK) for help with sample collection and processing. Finally, we thank Nathalie Smerdon, Richard Rance, Lucy Hildyard, Ben Softly, and Britt Killian for help with sample management, DNA sequencing, and data processing. G.S.V. is a consultant for KYMAB and receives an educational grant from Celgene.

Received: December 14, 2014
Revised: January 19, 2015
Accepted: January 29, 2015
Published: February 26, 2015

REFERENCES

Busque, L., Patel, J.P., Figueroa, M.E., Vasanthakumar, A., Provost, S., Hamilou, Z., Mollica, L., Li, J., Viale, A., Heguy, A., et al. (2012). Recurrent somatic TET2 mutations in normal elderly individuals with clonal hematopoiesis. Nat. Genet. *44*, 1179–1181.

Calvi, L.M., Adams, G.B., Weibrecht, K.W., Weber, J.M., Olson, D.P., Knight, M.C., Martin, R.P., Schipani, E., Divieti, P., Bringhurst, F.R., et al. (2003). Osteoblastic cells regulate the haematopoietic stem cell niche. Nature *425*, 841–846.

Cancer Genome Atlas Research Network (2013). Genomic and epigenomic landscapes of adult de novo acute myeloid leukemia. N. Engl. J. Med. *368*, 2059–2074.

Cazzola, M., Della Porta, M.G., and Malcovati, L. (2013). The genetic basis of myelodysplasia and its clinical relevance. Blood *122*, 4021–4034.

Chambers, S.M., Shaw, C.A., Gatza, C., Fisk, C.J., Donehower, L.A., and Goodell, M.A. (2007). Aging hematopoietic stem cells decline in function and exhibit epigenetic dysregulation. PLoS Biol. *5*, e201.

Conte, N., Varela, I., Grove, C., Manes, N., Yusa, K., Moreno, T., Segonds-Pichon, A., Bench, A., Gudgin, E., Herman, B., et al. (2013). Detailed molecular characterisation of acute myeloid leukaemia with a normal karyotype using targeted DNA capture. Leukemia *27*, 1820–1825.

Ding, L., Ley, T.J., Larson, D.E., Miller, C.A., Koboldt, D.C., Welch, J.S., Ritchey, J.K., Young, M.A., Lamprecht, T., McLellan, M.D., et al. (2012). Clonal evolution in relapsed acute myeloid leukaemia revealed by whole-genome sequencing. Nature *481*, 506–510.

Forbes, S.A., Beare, D., Gunasekaran, P., Leung, K., Bindal, N., Boutselakis, H., Ding, M., Bamford, S., Cole, C., Ward, S., et al. (2015). COSMIC: exploring the world's knowledge of somatic mutations in human cancer. Nucleic Acids Res. *43*, D805–D811.

Ford, A.M., Bennett, C.A., Price, C.M., Bruin, M.C., Van Wering, E.R., and Greaves, M. (1998). Fetal origins of the TEL-AML1 fusion gene in identical twins with leukemia. Proc. Natl. Acad. Sci. USA *95*, 4584–4588.

Fraga, M.F., Ballestar, E., Paz, M.F., Ropero, S., Setien, F., Ballestar, M.L., Heine-Suñer, D., Cigudosa, J.C., Urioste, M., Benitez, J., et al. (2005). Epigenetic differences arise during the lifetime of monozygotic twins. Proc. Natl. Acad. Sci. USA *102*, 10604–10609.

Genovese, G., Kähler, A.K., Handsaker, R.E., Lindberg, J., Rose, S.A., Bakhoum, S.F., Chambert, K., Mick, E., Neale, B.M., Fromer, M., et al. (2014). Clonal hematopoiesis and blood-cancer risk inferred from blood DNA sequence. N. Engl. J. Med. *371*, 2477–2487.

Greaves, M., and Maley, C.C. (2012). Clonal evolution in cancer. Nature *481*, 306–313.

Haferlach, T., Nagata, Y., Grossmann, V., Okuno, Y., Bacher, U., Nagae, G., Schnittger, S., Sanada, M., Kon, A., Alpermann, T., et al. (2014). Landscape of genetic lesions in 944 patients with myelodysplastic syndromes. Leukemia *28*, 241–247.

Holstege, H., Pfeiffer, W., Sie, D., Hulsman, M., Nicholas, T.J., Lee, C.C., Ross, T., Lin, J., Miller, M.A., Ylstra, B., et al. (2014). Somatic mutations found in the healthy blood compartment of a 115-yr-old woman demonstrate oligoclonal hematopoiesis. Genome Res. *24*, 733–742.

Jacobs, K.B., Yeager, M., Zhou, W., Wacholder, S., Wang, Z., Rodriguez-Santiago, B., Hutchinson, A., Deng, X., Liu, C., Horner, M.J., et al. (2012). Detectable clonal mosaicism and its relationship to aging and cancer. Nat. Genet. *44*, 651–658.

Jaiswal, S., Fontanillas, P., Flannick, J., Manning, A., Grauman, P.V., Mar, B.G., Lindsley, R.C., Mermel, C.H., Burtt, N., Chavez, A., et al. (2014). Age-related clonal hematopoiesis associated with adverse outcomes. N. Engl. J. Med. *371*, 2488–2498.

Krönke, J., Bullinger, L., Teleanu, V., Tschürtz, F., Gaidzik, V.I., Kühn, M.W., Rücker, F.G., Holzmann, K., Paschka, P., Kapp-Schwörer, S., et al. (2013). Clonal evolution in relapsed NPM1-mutated acute myeloid leukemia. Blood *122*, 100–108.

Kyle, R.A., Therneau, T.M., Rajkumar, S.V., Offord, J.R., Larson, D.R., Plevak, M.F., and Melton, L.J., 3rd. (2002). A long-term study of prognosis in monoclonal gammopathy of undetermined significance. N. Engl. J. Med. *346*, 564–569.

Laurie, C.C., Laurie, C.A., Rice, K., Doheny, K.F., Zelnick, L.R., McHugh, C.P., Ling, H., Hetrick, K.N., Pugh, E.W., Amos, C., et al. (2012). Detectable clonal mosaicism from birth to old age and its relationship to cancer. Nat. Genet. *44*, 642–650.

Li, H., Handsaker, B., Wysoker, A., Fennell, T., Ruan, J., Homer, N., Marth, G., Abecasis, G., and Durbin, R.; 1000 Genome Project Data Processing Subgroup (2009). The Sequence Alignment/Map format and SAMtools. Bioinformatics *25*, 2078–2079.

Lin, C.C., Hou, H.A., Chou, W.C., Kuo, Y.Y., Wu, S.J., Liu, C.Y., Chen, C.Y., Tseng, M.H., Huang, C.F., Lee, F.Y., et al. (2014). SF3B1 mutations in patients with myelodysplastic syndromes: the mutation is stable during disease evolution. Am. J. Hematol. *89*, E109–E115.

Matsunawa, M., Yamamoto, R., Sanada, M., Sato-Otsubo, A., Shiozawa, Y., Yoshida, K., Otsu, M., Shiraishi, Y., Miyano, S., Isono, K., et al. (2014). Haploinsufficiency of Sf3b1 leads to compromised stem cell function but not to myelodysplasia. Leukemia *28*, 1844–1850.

Nguyen-Dumont, T., Pope, B.J., Hammet, F., Southey, M.C., and Park, D.J. (2013). A high-plex PCR approach for massively parallel sequencing. Biotechniques *55*, 69–74.

Nowell, P.C. (1976). The clonal evolution of tumor cell populations. Science *194*, 23–28.

Papaemmanuil, E., Gerstung, M., Malcovati, L., Tauro, S., Gundem, G., Van Loo, P., Yoon, C.J., Ellis, P., Wedge, D.C., Pellagatti, A., et al.; Chronic Myeloid Disorders Working Group of the International Cancer Genome Consortium (2013). Clinical and biological implications of driver mutations in myelodysplastic syndromes. Blood *122*, 3616–3627, quiz 3699.

Rossi, D.J., Bryder, D., Zahn, J.M., Ahlenius, H., Sonu, R., Wagers, A.J., and Weissman, I.L. (2005). Cell intrinsic alterations underlie hematopoietic stem cell aging. Proc. Natl. Acad. Sci. USA *102*, 9194–9199.

Rossi, D.J., Jamieson, C.H., and Weissman, I.L. (2008). Stems cells and the pathways to aging and cancer. Cell *132*, 681–696.

Rübe, C.E., Fricke, A., Widmann, T.A., Fürst, T., Madry, H., Pfreundschuh, M., and Rübe, C. (2011). Accumulation of DNA damage in hematopoietic stem and progenitor cells during human aging. PLoS ONE *6*, e17487.

Shen, Z., Qu, W., Wang, W., Lu, Y., Wu, Y., Li, Z., Hang, X., Wang, X., Zhao, D., and Zhang, C. (2010). MPprimer: a program for reliable multiplex PCR primer design. BMC Bioinformatics *11*, 143.

Shlush, L.I., Zandi, S., Mitchell, A., Chen, W.C., Brandwein, J.M., Gupta, V., Kennedy, J.A., Schimmer, A.D., Schuh, A.C., Yee, K.W., et al.; HALT Pan-Leukemia Gene Panel Consortium (2014). Identification of pre-leukaemic haematopoietic stem cells in acute leukaemia. Nature *506*, 328–333.

Taraldsrud, E., Grøgaard, H.K., Solheim, S., Lunde, K., Fløisand, Y., Arnesen, H., Seljeflot, I., and Egeland, T. (2009). Age and stress related phenotypical changes in bone marrow CD34+ cells. Scand. J. Clin. Lab. Invest. *69*, 79–84.

Vas, V., Senger, K., Dörr, K., Niebel, A., and Geiger, H. (2012). Aging of the microenvironment influences clonality in hematopoiesis. PLoS ONE *7*, e42080.

Visconte, V., Rogers, H.J., Singh, J., Barnard, J., Bupathi, M., Traina, F., McMahon, J., Makishima, H., Szpurka, H., Jankowska, A., et al. (2012). SF3B1 haploinsufficiency leads to formation of ring sideroblasts in myelodysplastic syndromes. Blood *120*, 3173–3186.

Woolthuis, C.M., de Haan, G., and Huls, G. (2011). Aging of hematopoietic stem cells: Intrinsic changes or micro-environmental effects? Curr. Opin. Immunol. *23*, 512–517.

Wu, S.J., Kuo, Y.Y., Hou, H.A., Li, L.Y., Tseng, M.H., Huang, C.F., Lee, F.Y., Liu, M.C., Liu, C.W., Lin, C.T., et al. (2012). The clinical implication of SRSF2 mutation in patients with myelodysplastic syndrome and its stability during disease evolution. Blood *120*, 3106–3111.

Xie, M., Lu, C., Wang, J., McLellan, M.D., Johnson, K.J., Wendl, M.C., McMichael, J.F., Schmidt, H.K., Yellapantula, V., Miller, C.A., et al. (2014). Age-related mutations associated with clonal hematopoietic expansion and malignancies. Nat. Med. *20*, 1472–1478.

Zhang, J., Niu, C., Ye, L., Huang, H., He, X., Tong, W.G., Ross, J., Haug, J., Johnson, T., Feng, J.Q., et al. (2003). Identification of the haematopoietic stem cell niche and control of the niche size. Nature *425*, 836–841.

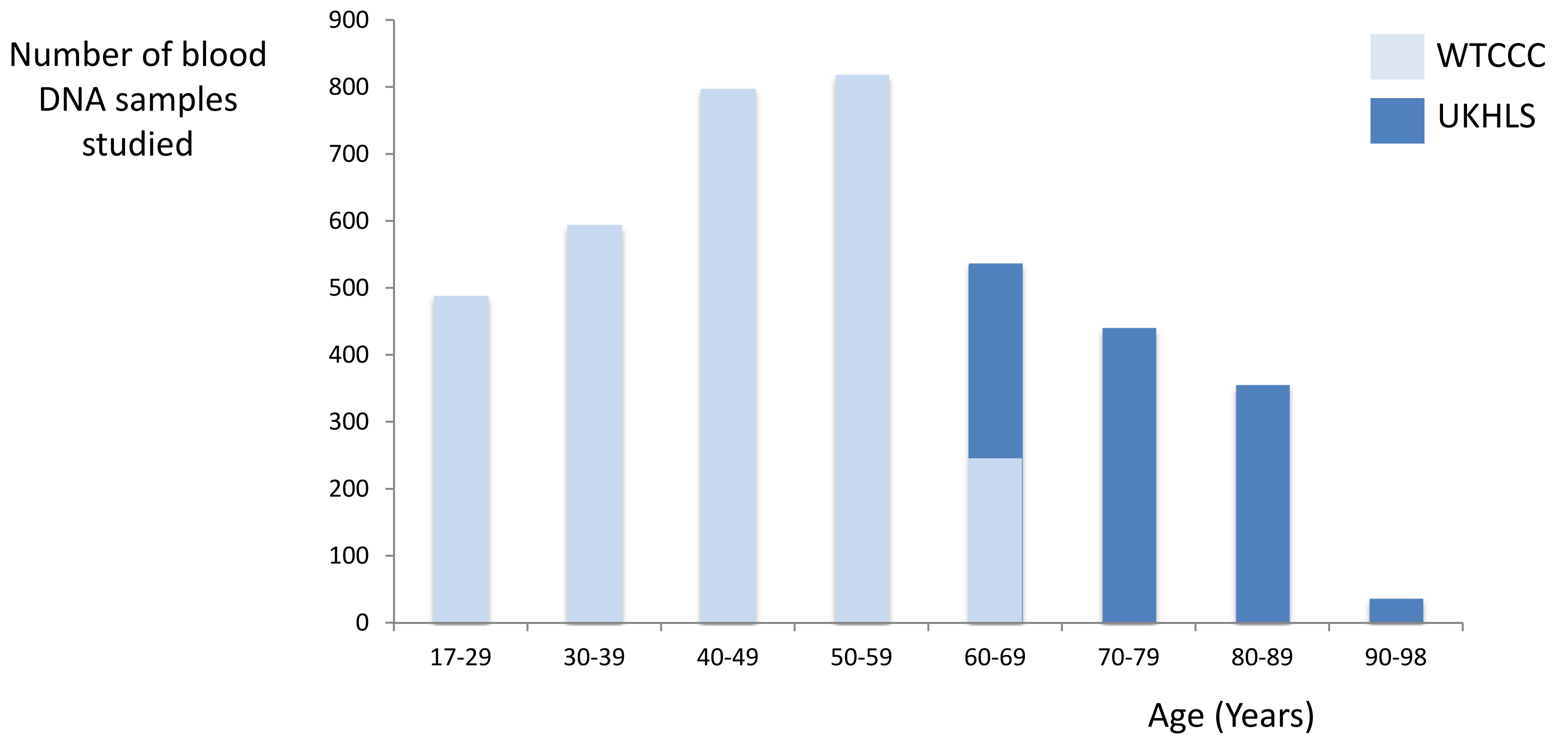
Number of blood DNA samples studied
900
800
700
600
500
400
300
200
100
0
17-29
30-39
40-49
50-59
60-69
70-79
80-89
90-98
Age (Years)
WTCCC
UKHLS

Figure S1

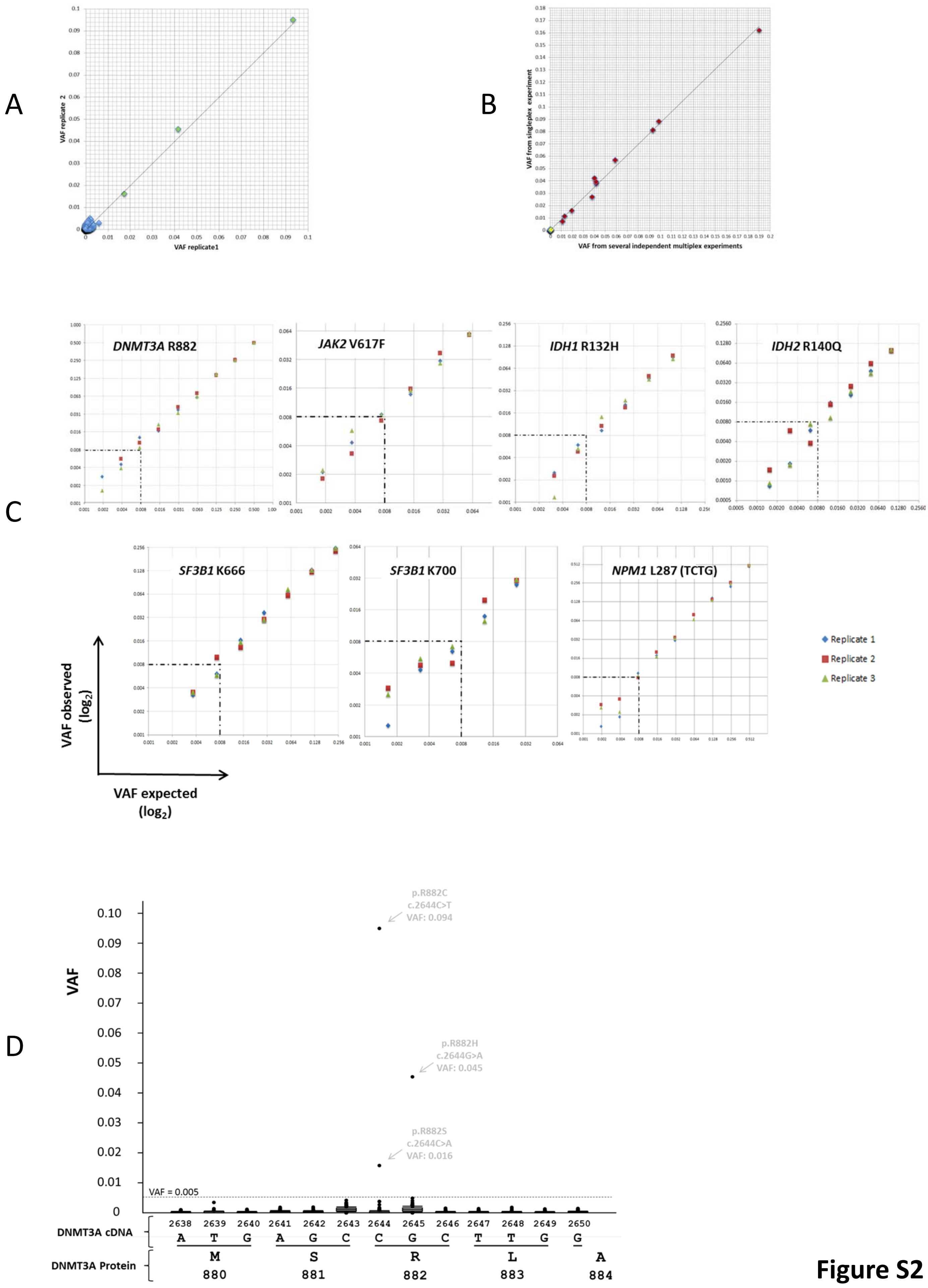
A
VAF replicate 2
VAF replicate1
B
VAF from singleplex experiment
VAF from several independent multiplex experiments
C
DNMT3A R882
JAK2 V617F
IDH1 R132H
IDH2 R140Q
SF3B1 K666
SF3B1 K700
NPM1 L287 (TCTG)
Replicate 1
Replicate 2
Replicate 3
VAF observed (log2)
VAF expected (log2)
D
VAF
p.R882C
c.2644C>T
VAF: 0.094
p.R882H
c.2644G>A
VAF: 0.045
p.R882S
c.2644C>A
VAF: 0.016
VAF = 0.005
DNMT3A cDNA
2638 2639 2640 2641 2642 2643 2644 2645 2646 2647 2648 2649 2650
A T G A G C C G C T T G G
DNMT3A Protein
M S R L A
880 881 882 883 884

Figure S2

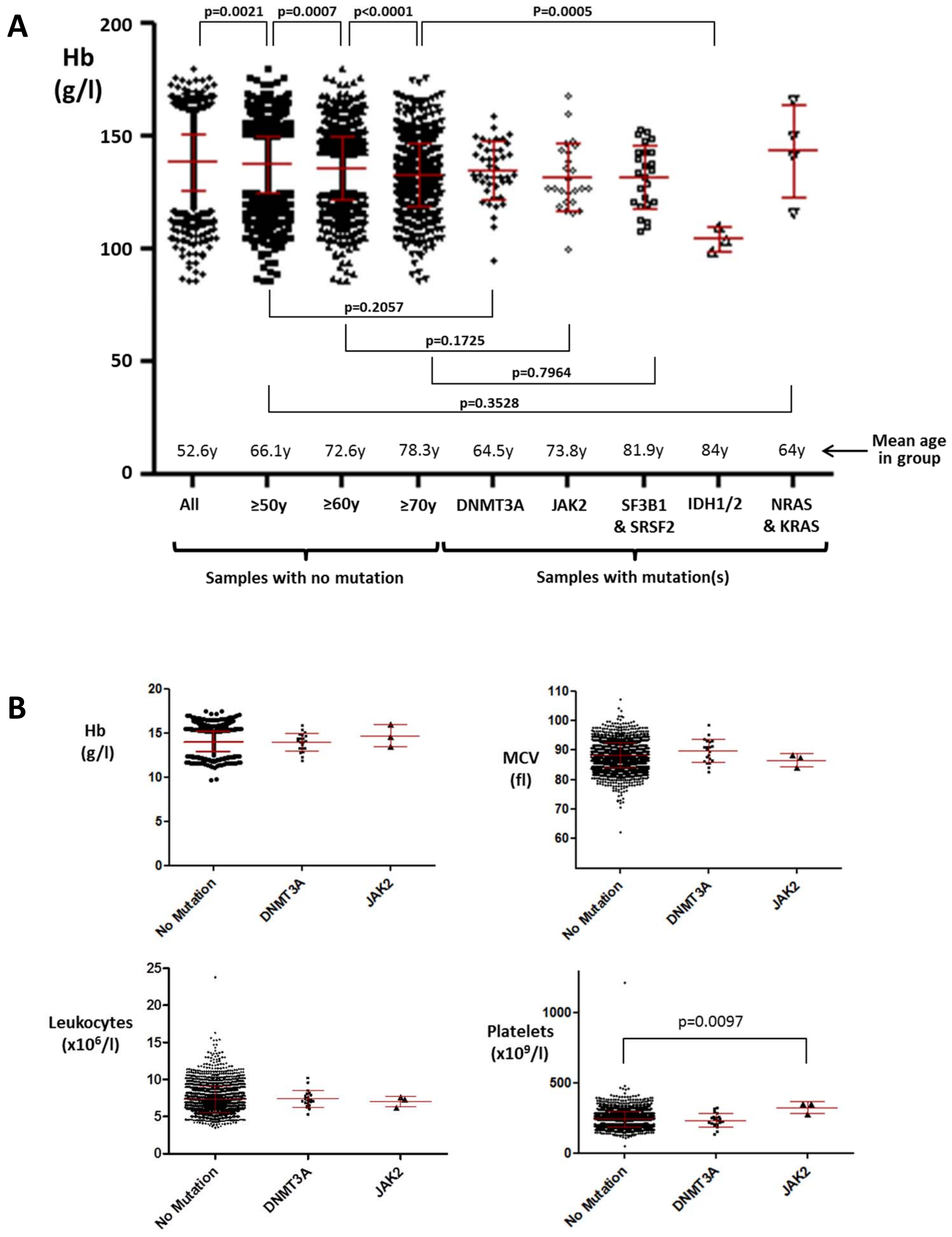
A
Hb
(g/l)
p=0.0021
p=0.0007
p<0.0001
P=0.0005
p=0.2057
p=0.1725
p=0.7964
p=0.3528
52.6y
66.1y
72.6y
78.3y
64.5y
73.8y
81.9y
84y
64y
Mean age in group
All
≥50y
≥60y
≥70y
DNMT3A
JAK2
SF3B1 & SRSF2
IDH1/2
NRAS & KRAS
Samples with no mutation
Samples with mutation(s)
B
Hb (g/l)
MCV (fl)
Leukocytes (x10⁶/l)
Platelets (x10⁹/l)
p=0.0097
No Mutation
DNMT3A
JAK2

Figure S3

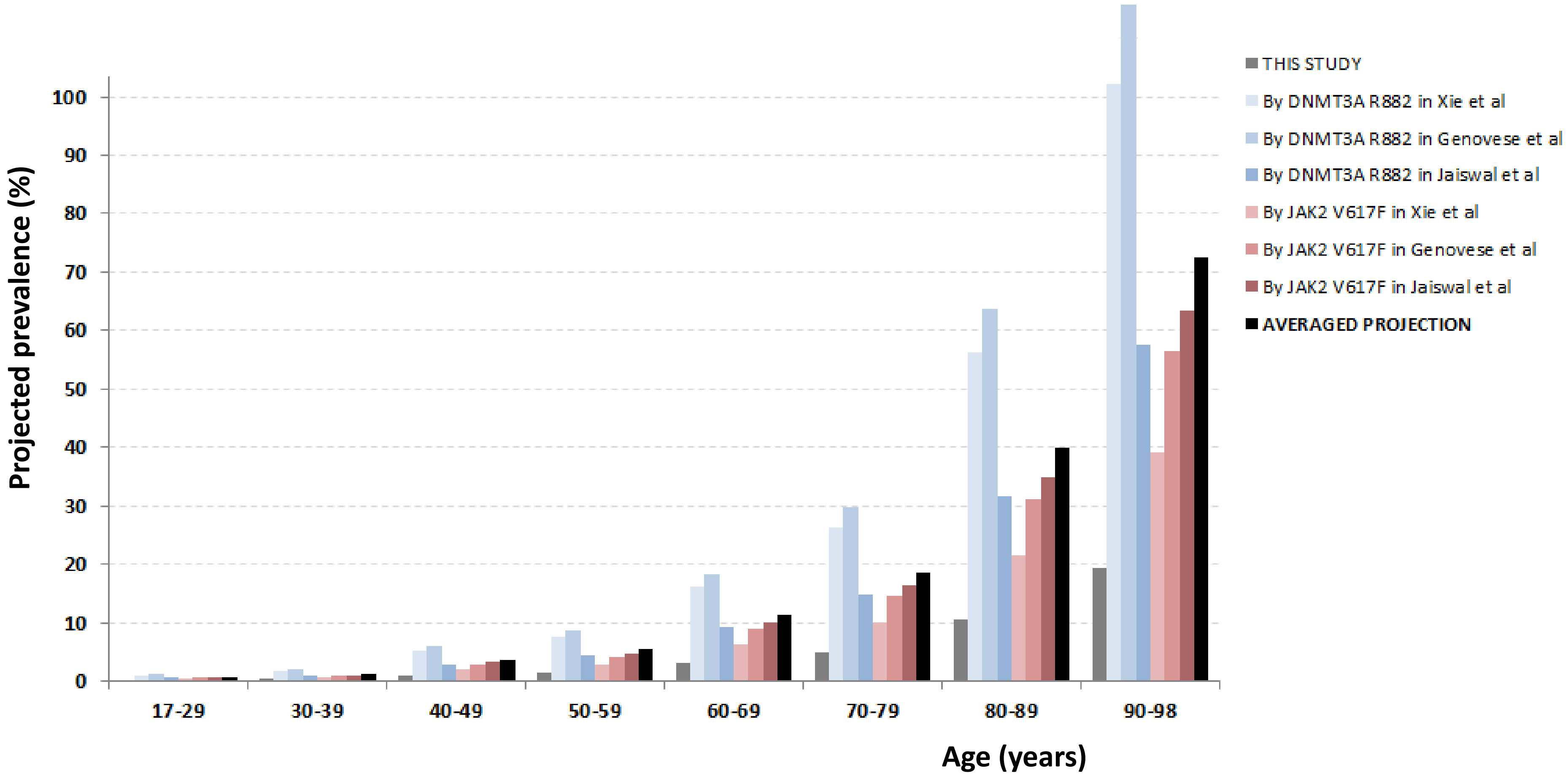
Projected prevalence (%)
100
90
80
70
60
50
40
30
20
10
0
17-29
30-39
40-49
50-59
60-69
70-79
80-89
90-98
Age (years)
THIS STUDY
By DNMT3A R882 in Xie et al
By DNMT3A R882 in Genovese et al
By DNMT3A R882 in Jaiswal et al
By JAK2 V617F in Xie et al
By JAK2 V617F in Genovese et al
By JAK2 V617F in Jaiswal et al
AVERAGED PROJECTION

Figure S4

SUPPLEMENTAL FIGURE LEGENDS

Figure S1. Numbers of individuals/DNA samples studied for the presence of clonal hemopoiesis

The sample group from which blood DNA samples were obtained is indicated. Samples were studied with different but overlapping sets of multiplex PCR primers (Plex 1-3, see Table S1). WTCC= Wellcome Trust Case Control Consortium, UKHLS= United Kingdom Household Longitudinal Study.

Related to experimental procedures

Figure S2. Methodological validation of multiplex PCR-MiSeq

A. To validate the reproducibility, accuracy and error rate of our multiplex PCR-MiSeq sequencing protocol, we studied the same 384 blood DNA samples twice in independent experiments and derived VAF values for the two *DNMT3A* R882 mutation hotspot positions, c.2644 and c.2645. The 371 samples with more than 1000 reads in both experiments are plotted (i.e. 2 replicates of 742 VAF datapoints). VAFs from the same sample and position from experiment 1 are plotted against the equivalent value from experiment 2. The 739 samples with VAFs ≤0.006 (blue diamonds) show relatively poor correlation in keeping with PCR/sequencing error at these very low VAF values. Nevertheless, no VAF values >0.006 are seen in either replicate except for the three real mutant samples (green diamonds) whose higher VAFs correlate extremely well**.**

B. To validate the reproducibility of our protocol for quantifying real mutations with VAFs ≥0.008, we re-analyzed 11 samples carrying subclonal DNA mutations with VAFs from 0.01 to 0.19 (i.e. 1-19% mutant reads) and 14 samples without evidence of subclonal mutations determined in several independent multiplex PCR experiments/batches , using an independent singleplex PCR for *DNMT3A* R882 (different primers). As shown, there was excellent correlation between multiplex and singleplex VAFs for all 11 mutant samples (red diamonds). The 14 samples without evidence of subclonal mutations according to the multiplex PCR, again gave very low VAFs (<0.0004) with singleplex analysis (yellow diamonds), therefore demonstrating the robust VAF quantitation achieved using our approach.

C. To validate the linearity of VAF quantitation of our protocol for the detection of low level subclonal mutations, we analyzed serial 2-fold dilutions of primary leukaemic or cell line (OCI-AML3) DNA into unmutated cord blood DNA for the following codons: *DNMT3A* R882, *JAK2* V617F, *IDH1* R132, *IDH2* R140, *SF3B1* K666, *SF3B1* K700 and *NPM1* L287. The “expected” VAF values were derived using the VAF obtained for the neat DNA of primary leukaemic or OCI-AML3 DNA in each experiment. Results of 3 independent replicate experiments using the same diluted DNA samples are shown. All hotspot loci studied show very good correlation between replicates for VAFs ≥0.008 (0.8%), our cut-off for “calling” mutant clones.

D. To derive the likelihood of obtaining a false positive mutation call we calculated the VAF for nucleotide positions surrounding the *DNMT3A* R882 codon outside of the mutation hotspots (c.2644 and c.2645) in 371 samples. Across all samples analyzed in this way, nucleotide positions outside the mutation hotspots gave VAFs lower than 0.005 (maximum VAF was 0.0041 at position c.2643). At the two hotspot positions we observed 3 samples with VAFs much higher than 0.005 (arrows). Near identical results were obtained when the same samples were analyzed for a second time using independent PCR amplifications and sequencing. These data were used to derive the likelihood of obtaining an erroneous VAF value greater than 0.008 (i.e. a false positive mutation call).

Related to experimental procedures

Figure S3. Comparisons of blood results between participants with different mutations

A. Hemoglobin concentrations (Hb) in different age and mutation groups for all participants (WTCCC & UKHLS). Individual values are plotted and red bars represent mean +/ ‐ standard deviation for each group. Paired t ‐ tests were performed for the indicated comparisons and, amongst individuals with no mutations, values were found to differ significantly between age groups (p values for each comparison indicated). Therefore, for each mutation group the “no mutation” group with the most similar mean age was used as control. Only IDH1/2 mutant samples had significantly lower Hb compared to age-matched controls. The 6 samples with more than one mutation were classified according to the mutation with the highest VAF.

B. Blood count results in different WTCCC participant groups. Individual values are plotted and red bars represent mean +/- standard deviation for each group. Paired t-tests were performed comparing “No mutation” samples with the DNMT3A mutant group (n=24) and, separately, with the JAK2 mutant group (n=3). The only significant comparison ($p<0.05$) was for platelet counts, which were higher in the JAK2 group. However, values for all 3 JAK2 mutant samples were within the reference range. The one KRAS and one NRAS mutant samples identified in the WTCCC sample group were not included in these comparisons. MCV=mean corpuscular volume (of erythrocytes).

Related to Figure 1

Figure S4. Projected overall prevalence of Age-Related Clonal Haemopoiesis driven by leukemia-associated mutations

Our methodology for detecting hotspot mutations was much more sensitive than approaches used by others to date. In order to derive approximate estimates of the overall prevalence of ARCH driven by leukemia-associated mutations, we projected our findings onto those of published datasets from three recent studies that used whole-exome sequencing of blood DNA to identify individuals with ARCH (Xi et al, 2014; Genovese et al; Jaiswal et al). As the age-distribution of participants varied significantly between studies and details of age-distribution of individuals mutations were not given by the two largest studies (Genovese et al; Jaiswal et al), we used the fraction of all mutations represented by DNMT3A R882 and by JAK2 V617F in each study to derive estimates of the overall prevalence of ARCH at the sensitivity of our study (i.e. VAF≥0.008). We chose these two mutations as they are the two commonest recurrent events in our study and also because they were identified in most age groups, albeit at varying frequencies. A notable limitation of this approach is the fact that exome sequencing detects different mutations with different sensitivities. In fact sequence coverage for DNMT3A R882 was lower, whilst that for JAK2 V617 was higher than average. Also each study set a different minimum VAF for “calling” mutations (Xie et al, VAF≥0.1; Genovese et al, VAF≥0.05 and Jaiswal et al, VAF≥0.03). Nevertheless, even the most conservative of our projections indicate that ARCH is much commoner than previously considered and is likely to occur in the majority of people aged over 90 years.

Related to Figure 1

Gene	Target codon	Plex 1	Plex 2	Plex 3	Numbers studied at each locus	Number of mutations per locus	Incidence of mutations per locus (%)
DNMT3A	R882	✓	✓	✓	4067	47	1.16
JAK2	V617	✓	✓	✓	4067	25	0.61
NPM1	L287	✓	✓	✓	4067	0	0.00
SRSF2	P95	✓		✓	2577	13	0.50
SF3B1	K666	✓		✓	2577	10	0.39
SF3B1	K700	✓		✓	2577	8	0.31
IDH1	R132	✓		✓	2577	2	0.08
IDH2	R140	✓		✓	2577	1	0.04
IDH2	R172	✓		✓	2577	0	0.00
KRAS	G12		✓	✓	2606	2	0.08
NRAS	G12		✓	✓	2606	2	0.08
NRAS	Q61		✓	✓	2606	0	0.00
KIT	D816		✓	✓	2606	0	0.00
FLT3	D835		✓	✓	2606	0	0.00
FLT3	N676		✓		1490	0	0.00
Number of individuals screened using each Plex design		1531	1536	1152			
Number of individuals with adequate coverage for analysis*		1461	1490	1116			

Table S1

Study	Mutation Group	Mutation number	Read depth (coverage)*			
			Mean	SD**	Minimum	Maximum
Genovese et al, 2014	All mutations	327	91.7	51.0	11	371
	DNMT3A (all)	190	91.2	49.2	21	255
	DNMT3A R882	123	58.6	17.2	30	101
	JAK2 V617F	24	111.0	27.1	79	191
	SF3B1 K666	3	162.0	89.3	72	234
	SF3B1 K700	9	89.3	32.7	59	164
	SRSF2P95	5	59.0	10.3	47	69
Jaiswal et al, 2014	All mutations	805	94.0	53.9	16	432
	DNMT3A (all)	403	92.6	50.7	18	384
	DNMT3A R882	67	61.9	15.9	34	95
	JAK2 V617F	31	121.8	38.8	81	265
	SF3B1 K666	11	69.5	15.7	52	107
	SF3B1K700	12	82.5	26.7	38	133
	SRSF2 P95	10	51.8	11.3	35	72
Xie et al, 2014	All mutations	77	107.8	69.0	22	387
	DNMT3A (all)	18	109.3	86.8	28	387
	DNMT3A R882	6	65.5	31.6	28	115
	JAK2 V617	8	156.0	66.0	63	237
	SF3B1K666	0	n/a	n/a	n/a	n/a
	SF3B1K700	2	90.0	15.6	79	101
	SRSF2 P95	0	n/a	n/a	n/a	n/a

Table S2

Patient age	Gender	Indication for autologous HSCT	Time since HSCT (months)
61	M	Myeloma	12
68	M	Hodgkins Lymphoma	4.5
63	M	Mantle Cell Lymphoma	1
27	M	Hodgkins Lymphoma	17
53	F	Follicular Lymphoma	8
41	F	Diffuse Large B Cell Lymphoma	12
66	F	Mantle cell Lymphoma	24
63	M	Myeloma	34
57	F	Diffuse Large B Cell Lymphoma	15
55	M	Myeloma	26
51	M	Myeloma	65
49	M	NK T cell Lymphoma	15

Table S3

Patient age	Gender	Indication for Allogeneic HSCT	Time since HSCT (months)	Donor age at sampling	Donor Gender	Donor Chimerism (%)
63	M	Diffuse Large B Cell Lymphoma	5	67	F	95
52	F	Chronic Lymphocytic Leukemia	36	Unknown	M	99
52	M	Angioimmunoblastic lymphoma	18	47	F	100
61	M	Myelodysplastic syndrome (RAEB)	63	27	M	100
33	M	Non-Hodgkin's Lymphoma	94	37	F	Unknown
58	M	Acute Myeloid Leukemia	15	Unknown	M	100
59	M	Acute Myeloid Leukemia	25	Unknown	M	100
44	F	Acute Myeloid Leukemia	34	Unknown	F	100
41	M	Myeloma	22	Unknown	M	100
49	M	Acute Myeloid Leukemia	105	Unknown	M	Unknown
56	F	Acute Myeloid Leukemia	63	42	M	96
47	F	Chronic Myeloid Leukemia	170	Unknown	Unknown	Unknown
50	M	Blast crisis of Chronic Myeloid Leukemia	30	Unknown	M	Unknown
19	M	Aplastic Anaemia	13	15	F	98
67	M	Secondary Acute Myeloid Leukemia	45	Unknown	M	100
25	M	Hodgkin's Lymphoma	42	27	F	100
65	F	Acute Myeloid Leukemia	62	44	M	99
61	M	Acute Myeloid Leukemia	13	Unknown	M	100
58	F	Secondary Acute Myeloid Leukemia	14	Unknown	M	100
48	F	Acute Lymphoblastic Leukemia	62	58	M	100

Table S4

SUPPLEMENTAL TABLE LEGENDS

Table S1. Multiplex PCR reactions and numbers of individuals (blood DNA samples) studied

WTCCC samples were screened with Plex 1 or Plex2. UKHLS samples were screened with Plex 3

* Only samples with ≥1000 reads at all studied loci were interrogated for the presence of mutations

Related to Table 1

Table S2. Read depth statistics for selected mutation calls in three recent studies using exome sequencing to identify individuals with ARCH

* Total read count (reference reads + mutant reads)

** SD = standard deviation

NB: These statistics are for coverage at called mutations. Numerical read depth (coverage) values for samples without identified mutations at these loci were not provided.

Related to Figure 1 & Table 2

Table S3. Characteristics of individuals sampled after autologous hematopoietic stem cell transplantation (HSCT)

Related to experimental procedures

Table S4. Characteristics of individuals sampled after allogeneic hematopoietic stem cell transplantation (HSCT) and their respective donors

Related to experimental procedures

Supplemental Experimental Procedures

Targeted re-sequencing

Multiplex primer combinations were tested and their concentrations adjusted to give similar levels of amplification for each of the target positions. First round multiplex PCR amplifications were performed with tailed gene primers on batches of up to 384 samples and individually barcoded by second round PCR with 384 pre-validated "MiSeq-ready" primers[1]; using a high fidelity polymerase (KAPA HiFi, Anachem or Q5 Hot Start HF, New England Biolabs). PCR reaction conditions used were as follows: 95°C for 3min, [98°C for 20s, 65°C for 60s, 60°C for 60s, 55°C for 60s, 50°C for 60s, 70°C for 60s] x6 cycles, held at 4°C until addition of barcoded second round primers, then [98°C for 20s, 62°C for 15s, 72°C for 30s] x19 cycles, 72°C 60s. For each batch, equal volumes of each sample were pooled, double SPRI size selected (X0.55 and X0.75) and quantified before storage at -20°C until sequencing. A total of 11 MiSeq runs (250nt paired-end) were used for mutation identification. One of the 11 sample batches was repeated from PCR to sequencing for experimental validation purposes (Supplemental Figure S2A). Also, the reproducibility of our assay in quantifying variant allele fractions (VAFs) was further confirmed by studying 11 unselected samples harboring *DNMT3A*-R882 mutant clones of varying sizes (VAF 0.01-0.18), using a different *DNMT3A*-R882 primer set in a singleplex PCR using the following conditions: 98° for 30s [98° for 20s, 60oC for 30s, 72°C for 60s] x6 cycles, held at 4oC until addition of barcoded second round primers, then [98°C for 20s, 62°C for 15s, 72°C for 30s] x19 cycles, 72°C 60s (Supplemental Figure S2B). Finally, the linearity of VAF calling was confirmed, using serial dilutions of leukemia or cell line DNA into cord blood DNA, for specific mutations including *DNMT3A*-R882, *JAK2*-V617F, *IDH1*-R132H, *IDH2*-R140Q, *SF3B1*-K666N, *SF3B1*-K700 and *NPM1*_mutation_A (TCTG duplication) (Supplemental figures S2C). The first 1571 samples (1531 WTCC, 32 post-transplant and 18 cord blood) were amplified using "Plex 1", the next 1554 samples (1536 WTCC, and 18 cord blood) using "Plex 2" and the final 1152 samples (UKHLS) using Plex 3 primer sets.

Samples with two mutations at the same or neighboring loci

Amongst all samples, we identified 5 individuals with two independent spliceosome gene mutations of different VAFs (Figure 1B), 3 of which harbored the mutations at the same or at neighboring loci. Two of these, #760 and #565, harbored *SRSF2* P95H and *SRSF2* P95L, and another, #424, harbored *SF3B1* K666 and *SF3B1* K700. In an attempt to determine whether mutations were acquired on the same or on different alleles (maternal vs paternal), we looked for neighboring SNPs that could be used to "phase" the variants. We searched the Ensembl database for SNPs near *SRSF2* P95 and *SF3B1* K666/K700 and identified nearby polymorphisms for both locations, namely rs237057 (A/G MAF(G)=0.19) near *SRSF2* P95 and rs113023355 (A/G MAF(G)=0.012) near *SF3B1* K666 and K700. However, all three individuals were homozygous for the common alleles (A/A) and regrettably we were unable to phase the somatic variants.

Bioinformatic Analysis – Perl script for detecting *NPM1* mutations

In order to detect *NPM1* mutations with high sensitivity, we wrote a new Perl script to extract from each sample the reads covering the *NPM1* mutation hotspot and align these against the reference genome. Subsequently, the script individually parses each read looking for insertions at the hotspot position. The number of reads reporting the reference is recorded and so is the number reporting any variants and the sequence of this variant. Using sequencing data from normal samples manually spiked with *NPM1*-mutant DNA (OCI-AML3 cell line, mutation A), we determined that mutant reads with an expected VAF ≥ 0.002 (0.2%) were reliably detected (Supplemental Figure S2C).

Statistical Analysis and mutation calling threshold

We observed an apparent sequencing + PCR error rate < 0.13% after quality filters, which is broadly in line with sequencing errors observed elsewhere with current Illumina sequencing pipelines[2] and corresponds to a phred-scaled base call quality of 30. Postulating a binomial distribution of variant allele counts with this error probability and a total allele count (read depth) ≥ 1000, one would expect a false positive call rate $<10^{-5}$ when calling variants at VAF ≥ 0.008. To test this, we analyzed the range of VAFs derived from the study of 384 WTCC samples at 13 nucleotide positions at and around *DNMT3A* codon R882. Only amplicons giving ≥1000 reads were included in analyses. We found that the 3710 VAF values (10 positions x 371 samples) at positions outside the R882 hotspot (i.e. non-targets of known leukemia-associated mutations) were always ≤0.0045 indicating a very small combined PCR-MiSeq error rate. The 3 real subclonal samples in this group of 384 were easily distinguishable from error (Supplemental Figure S2D)

Supplemental References

1 Quail, M. A. *et al.* SASI-Seq: sample assurance Spike-Ins, and highly differentiating 384 barcoding for Illumina sequencing. *BMC genomics* **15**, 110, doi:10.1186/1471-2164-15-110 (2014).

2 Ekblom, R., Smeds, L. & Ellegren, H. Patterns of sequencing coverage bias revealed by ultra-deep sequencing of vertebrate mitochondria. *BMC genomics* **15**, 467, doi:10.1186/1471-2164-15-467 (2014).

Primer Name	Chromosome	Start coordinate (GRCh37)	Primer Sequence†
5247756-DNMT3A_p.R882_F	2	25457060	ACA CTC TTT CCC TAC ACG ACG CTC TTC CGA TCT CCT CAT GTT CTT GGT GTT TTmA T ‡
5247757-DNMT3A_p.R882_R	2	25457302	TCG GCA TTC CTG CTG AAC CGC TCT TCC GAT CTT TTT CTC CCC CAG GGT MTT mUG
5247759-IDH1_p.R132_F	2	209112927	ACA CTC TTT CCC TAC ACG ACG CTC TTC CGA TCT TAA ATG TGT GTA AAT ATA CAG TTmA T
5247760-IDH1_p.R132_R	2	209113173	TCG GCA TTC CTG CTG AAC CGC TCT TCC GAT CTR TTA TCT GCA AAA ATA TCY CmCC
5247762-IDH2_p.R140_R172_F	15	90631745	ACA CTC TTT CCC TAC ACG ACG CTC TTC CGA TCT AAG ARG ATG KCT AGG YGA GmGA
5247764-IDH2_p.R140_R172_R	15	90631986	TCG GCA TTC CTG CTG AAC CGC TCT TCC GAT CTC TCA MAG AGT TCA AGC TGA mAG
5247766-SRSF2_p.P95_F	17	74732797	ACA CTC TTT CCC TAC ACG ACG CTC TTC CGA TCT TGC TTC GCC GCG GAC CTT TmGT
5247767-SRSF2_p.P95_R	17	74733038	TCG GCA TTC CTG CTG AAC CGC TCT TCC GAT CTG AGG ACG CTA TGG ATG CCA mUG
5247768-SF3B1_p.K700_F	2	198266642	ACA CTC TTT CCC TAC ACG ACG CTC TTC CGA TCT TAG TAA TTT AGA TTT ATG TCG mCC
5247769-SF3B1_p.K700_R	2	198266886	TCG GCA TTC CTG CTG AAC CGC TCT TCC GAT CTG GCA TAG TTA AAA CCT GTG TmUT
5247770-SF3B1_p.K666_F	2	198267228	ACA CTC TTT CCC TAC ACG ACG CTC TTC CGA TCT ACC CTG TCT CCT AAA GAA AAmA A
5247771-SF3B1_p.K666_R	2	198267470	TCG GCA TTC CTG CTG AAC CGC TCT TCC GAT CTT AGA GCT TTT GCT GTT GTA mGC
5247772-NPM1_p.L287fsX_F	5	170837352	ACA CTC TTT CCC TAC ACG ACG CTC TTC CGA TCT TGT TTG GAA TTA AAT TAC ATC TmGA
5247773-NPM1_p.L287fsX_R	5	170837602	TCG GCA TTC CTG CTG AAC CGC TCT TCC GAT CTA AAA TTT TTT AAC AAA TTG TTT AAA mCT
5247774-repeat_CAG_F1	X	67545198	ACA CTC TTT CCC TAC ACG ACG CTC TTC CGA TCT AGG TGG ACC AGA AAT GGA AmAT
5247775-repeat_CAG_R1	X	67545441	TCG GCA TTC CTG CTG AAC CGC TCT TCC GAT CTT GTG GTC TTT ATC CAA AAG TTmU A
5739576-JAK2V617_F	9	5073696	ACA CTC TTT CCC TAC ACG ACG CTC TTC CGA TCT AGT CTT TCT TTG AAG CAG CAmA G
5739764-JAK2V617_R	9	5073887	TCG GCA TTC CTG CTG AAC CGC TCT TCC GAT CTA GTT TAC ACT GAC ACC TAG CmUG

Nucleotide sequences for multiplexed primers used in Plex 1

* Consecutive primers constitute forward (F) and reverse (R) primer pairs for the indicated loci

† Forward primers format: 5’ ACACTCTTTCCCTACACGACGCTCTTCCGATCT-[gene-specific forward] 3’, Reverse primerformat:5’ TCGGCATTCCTGCTGAACCGCTCTTCCGATCT-[gene-specific reverse] 3’

‡ "m" denotes a single 2'-O-Methyl base in place of the DNA base, used in order to minimise potential primer dimers

Primer Name*	Chromosome	Start coordinate (GRCh37)	Primer Sequence†
6029105-JAK2_V617_F	9	5073696	ACA CTC TTT CCC TAC ACG ACG CTC TTC CGA TCT AGT CTT TCT TTG AAG CAG CAmA G ‡
6029106-JAK2_V617_R	9	5073887	TCG GCA TTC CTG CTG AAC CGC TCT TCC GAT CTA GTT TAC ACT GAC ACC TAG CmUG
6029123-DNMT3A_R882_F	2	25457051	ACA CTC TTT CCC TAC ACG ACG CTC TTC CGA TCT TCT CTC CAT CCT CAT GTT CTmU G
6029124-DNMT3A_R882_R	2	25457284	TCG GCA TTC CTG CTG AAC CGC TCT TCC GAT CTT TGG TTT CCC AGT CCA CTA TmAC
6029109-TET2_H880_F	4	106157575	ACA CTC TTT CCC TAC ACG ACG CTC TTC CGA TCT AGT GCA TGC AAA ATA CAG GTmU T
6029110-TET2_H880_R	4	106157784	TCG GCA TTC CTG CTG AAC CGC TCT TCC GAT CTA ACT GAA GCT TGT TGT RAC TmUC
6029111-TET2_R1214_F	4	106164665	ACA CTC TTT CCC TAC ACG ACG CTC TTC CGA TCT TGA CCC TTG TTT TGT TTT GGmU T
6029112-TET2_R1214_F	4	106164877	TCG GCA TTC CTG CTG AAC CGC TCT TCC GAT CTT AAG CTC CGA GTA GAG TTT GmUC
6029113-KIT_exon8_F	4	55589690	ACA CTC TTT CCC TAC ACG ACG CTC TTC CGA TCT AGT GAA TGT TGC TGA GGT TTmU C
6029114-KIT_exon8_R	4	55589911	TCG GCA TTC CTG CTG AAC CGC TCT TCC GAT CTG TCC TTC CCC TCT GCA TTA TmAA
6029103-KIT_exon17_F	4	55599207	ACA CTC TTT CCC TAC ACG ACG CTC TTC CGA TCT TGG TTT TCT TTT CTC CTC CAmA C
6029104-KIT_exon17_R	4	55599396	TCG GCA TTC CTG CTG AAC CGC TCT TCC GAT CTT CCT TTG CAG GAC WGT CAmA G
6029115-NRAS_G12_F	1	115258606	ACA CTC TTT CCC TAC ACG ACG CTC TTC CGA TCT ATG GGT AAA GAT GAT CCG ACmA A
6029116-NRAS_G12_R	1	115258831	TCG GCA TTC CTG CTG AAC CGC TCT TCC GAT CTC GCC AAT TAA CCC TGA TTA CmUG
6029121-NRAS_Q61_F	1	115256340	ACA CTC TTT CCC TAC ACG ACG CTC TTC CGA TCT CCC TAG TGT GGT AAC CTC ATmU T
6029122-NRAS_Q61_R	1	115256573	TCG GCA TTC CTG CTG AAC CGC TCT TCC GAT CTA GAT GGT GAA ACC TGT TTG TmUR
6029107-KRAS_G12_F	12	25398214	ACA CTC TTT CCC TAC ACG ACG CTC TTC CGA TCT TGT TSG ATC ATA TTC RTC CAmC A
6029108-KRAS_G12_R	12	25398416	TCG GCA TTC CTG CTG AAC CGC TCT TCC GAT CTA AGG TAC TGG TGG AGT ATT TmGA
6029117-TET2_exon8_F	4	106182816	ACA CTC TTT CCC TAC ACG ACG CTC TTC CGA TCT TGG GAT TCA AAA TGT AAG GGmG A
6029118-TET2_exon8_R	4	106183041	TCG GCA TTC CTG CTG AAC CGC TCT TCC GAT CTT TGT TAC AAT TGC TGC CAA TmGA
6029119-FLT3_N676_F	13	28602158	ACA CTC TTT CCC TAC ACG ACG CTC TTC CGA TCT TGC TCA GTG TCT AAT TCC ACmU T
6029120-FLT3_N676_R	13	28602388	TCG GCA TTC CTG CTG AAC CGC TCT TCC GAT CTA GAA CTC AAG ATG ATG ACC CmAG
6029125-FLT3_D835_F	13	28592585	ACA CTC TTT CCC TAC ACG ACG CTC TTC CGA TCT TAG GAA ATA GCA GCC TCA CAmU T
6029126-FLT3_D835_F	13	28592819	TCG GCA TTC CTG CTG AAC CGC TCT TCC GAT CTG GTA CCT CCT ACT GAA GTT GmAG
6029127-ASXL1_F	20	31022393	ACA CTC TTT CCC TAC ACG ACG CTC TTC CGA TCT GGC GAG AGG TCA CCA CmUG
6029128-ASXL1_R	20	31022630	TCG GCA TTC CTG CTG AAC CGC TCT TCC GAT CTC TCC CYA TTT AGA GGA TAA GmGC
6029129-RUNX1_F	21	36252791	ACA CTC TTT CCC TAC ACG ACG CTC TTC CGA TCT TTT TGA AAT GTG GGT TTG TTmG C
6029130-RUNX1_R	21	36253035	TCG GCA TTC CTG CTG AAC CGC TCT TCC GAT CTC ATT TGT CCT TTG ACT GGT GmUT
NPM1_p.L287fsX_F	5	170837352	ACA CTC TTT CCC TAC ACG ACG CTC TTC CGA TCT TGT TTG GAA TTA AAT TAC ATC TmGA
NPM1_p.L287fsX_R	5	170837602	TCG GCA TTC CTG CTG AAC CGC TCT TCC GAT CTA AAA TTT TTT AAC AAA TTG TTT AAA mCT

Nucleotide sequences for multiplexed primers used in Plex 2

* Consecutive primers constitute forward (F) and reverse (R) primer pairs for the indicated loci

† Forward primers format: 5’ ACACTCTTTCCCTACACGACGCTCTTCCGATCT-[gene-specific forward] 3’, Reverse primerformat:5’ TCGGCATTCCTGCTGAACCGCTCTTCCGATCT-[gene-specific reverse] 3’

‡ "m" denotes a single 2'-O-Methyl base in place of the DNA base, used in order to minimise potential primer dimers

Primer Name*	Chromosome	Start coordinate (GRCh37)	Primer Sequence†
5247756-DNMT3A_p.R882_F	2	25457060	ACA CTC TTT CCC TAC ACG ACG CTC TTC CGA TCT CCT CAT GTT CTT GGT GTT TTmA T ‡
5247757-DNMT3A_p.R882_R	2	25457302	TCG GCA TTC CTG CTG AAC CGC TCT TCC GAT CTT TTT CTC CCC CAG GGT MTT mUG
5247759-IDH1_p.R132H_1_F	2	209112927	ACA CTC TTT CCC TAC ACG ACG CTC TTC CGA TCT TAA ATG TGT GTA AAT ATA CAG TTmA T
5247760-IDH1_p.R132H_1_R	2	209113173	TCG GCA TTC CTG CTG AAC CGC TCT TCC GAT CTR TTA TCT GCA AAA ATA TCY CmCC
5247762-IDH2_p.R140_R172_F	15	90631745	ACA CTC TTT CCC TAC ACG ACG CTC TTC CGA TCT AAG ARG ATG KCT AGG YGA GmGA
5247764-IDH2_p.R140_R172_R	15	90631986	TCG GCA TTC CTG CTG AAC CGC TCT TCC GAT CTC TCA MAG AGT TCA AGC TGA mAG
5247766-SRSF2_p.P95_F	17	74732797	ACA CTC TTT CCC TAC ACG ACG CTC TTC CGA TCT TGC TTC GCC GCG GAC CTT TmGT
5247767-SRSF2_p.P95_R	17	74733038	TCG GCA TTC CTG CTG AAC CGC TCT TCC GAT CTG AGG ACG CTA TGG ATG CCA mUG
5247768-SF3B1_p.K700_F	2	198266642	ACA CTC TTT CCC TAC ACG ACG CTC TTC CGA TCT TAG TAA TTT AGA TTT ATG TCG mCC
5247769-SF3B1_p.K700_R	2	198266886	TCG GCA TTC CTG CTG AAC CGC TCT TCC GAT CTG GCA TAG TTA AAA CCT GTG TmUT
5247770-SF3B1_p.K666_F	2	198267228	ACA CTC TTT CCC TAC ACG ACG CTC TTC CGA TCT ACC CTG TCT CCT AAA GAA AAmA A
5247771-SF3B1_p.K666_R	2	198267470	TCG GCA TTC CTG CTG AAC CGC TCT TCC GAT CTT AGA GCT TTT GCT GTT GTA mGC
5247772-NPM1_p.L287fsX_F	5	170837352	ACA CTC TTT CCC TAC ACG ACG CTC TTC CGA TCT TGT TTG GAA TTA AAT TAC ATC TmGA
5247773-NPM1_p.L287fsX_R	5	170837602	TCG GCA TTC CTG CTG AAC CGC TCT TCC GAT CTA AAA TTT TTT AAC AAA TTG TTT AAA mCT
5739576-JAK2V617_F	9	5073696	ACA CTC TTT CCC TAC ACG ACG CTC TTC CGA TCT AGT CTT TCT TTG AAG CAG CAmA G
5739764-JAK2V617_R	9	5073887	TCG GCA TTC CTG CTG AAC CGC TCT TCC GAT CTA GTT TAC ACT GAC ACC TAG CmUG
6029103-KIT_exon17_F	4	55599207	ACA CTC TTT CCC TAC ACG ACG CTC TTC CGA TCT TGG TTT TCT TTT CTC CTC CAmA C
6029104-KIT_exon17_R	4	55599396	TCG GCA TTC CTG CTG AAC CGC TCT TCC GAT CTT CCT TTG CAG GAC WGT CAmA G
6029107-KRAS_G12_F	12	25398214	ACA CTC TTT CCC TAC ACG ACG CTC TTC CGA TCT TGT TSG ATC ATA TTC RTC CAmC A
6029108-KRAS_G12_R	12	25398416	TCG GCA TTC CTG CTG AAC CGC TCT TCC GAT CTA AGG TAC TGG TGG AGT ATT TmGA
6029115-NRAS_G12_F	1	115258606	ACA CTC TTT CCC TAC ACG ACG CTC TTC CGA TCT ATG GGT AAA GAT GAT CCG ACmA A
6029116-NRAS_G12_R	1	115258831	TCG GCA TTC CTG CTG AAC CGC TCT TCC GAT CTC GCC AAT TAA CCC TGA TTA CmUG
6029121-NRAS_Q61_F	1	115256340	ACA CTC TTT CCC TAC ACG ACG CTC TTC CGA TCT CCC TAG TGT GGT AAC CTC ATmU T
6029122-NRAS_Q61_R	1	115256573	TCG GCA TTC CTG CTG AAC CGC TCT TCC GAT CTA GAT GGT GAA ACC TGT TTG TmUR
6029125-FLT3_D835_F	13	28592585	ACA CTC TTT CCC TAC ACG ACG CTC TTC CGA TCT TAG GAA ATA GCA GCC TCA CAmU T
6029126-FLT3_D835_R	13	28592819	TCG GCA TTC CTG CTG AAC CGC TCT TCC GAT CTG GTA CCT CCT ACT GAA GTT GmAG

Nucleotide sequences for multiplexed primers used in Plex 3

* Consecutive primers constitute forward (F) and reverse (R) primer pairs for the indicated loci

† Forward primers format: 5’ ACACTCTTTCCCTACACGACGCTCTTCCGATCT-[gene-specific forward] 3’, Reverse primerformat:5’ TCGGCATTCCTGCTGAACCGCTCTTCCGATCT-[gene-specific reverse] 3’

‡ "m" denotes a single 2'-O-Methyl base in place of the DNA base, used in order to minimise potential primer dimers
